# Generation of a genetically engineered porcine melanoma model featuring oncogenic control through conditional Cre recombination

**DOI:** 10.1038/s41598-024-82554-w

**Published:** 2025-01-10

**Authors:** Dongjin Oh, Nayoung Hong, Kiyoung Eun, Joohyeong Lee, Lian Cai, Mirae Kim, Hyerin Choi, Ali Jawad, Jaehyung Ham, Min Gi Park, Bohye Kim, Sang Chul Lee, Changjong Moon, Hyunggee Kim, Sang-Hwan Hyun

**Affiliations:** 1 Laboratory of Veterinary Embryology and Biotechnology (VETEMBIO), Veterinary Medical Center and College of Veterinary Medicine, Chungbuk National University, Cheongju, Republic of Korea; 2 Institute of Stem Cell and Regenerative Medicine (ISCRM), Chungbuk National University, Cheongju, Republic of Korea; 3 Department of Biotechnology, College of Life Sciences and Biotechnology, Korea University, Seoul, 02841 Republic of Korea; 4 Insitute of Animal Molecular Biotechnology, Korea University, Seoul, 02841 Republic of Korea; 5 Department of Hematology and Medical Oncology, Winship Cancer Institute of Emory, Emory University School of Medicine, Atlanta, GA 30322 USA; 6 Department of Companion Animal Industry, Semyung University, Jecheon, 27136 Republic of Korea; 7 Department of Veterinary Anatomy and Animal Behavior, College of Veterinary Medicine and BK21 FOUR Program, Chonnam National University, Gwangju, Republic of Korea; 8 Cronex Inc., Cheongju, 28174 Republic of Korea; 9 Vet-ICT Convergence Education and Research Center (VICERC), Chungbuk National University, Cheongju, Republic of Korea; 10 Chungbuk National University Hospital, Cheongju, Republic of Korea

**Keywords:** Melanoma, Transgenic cancer model, Pig, Tissue-specific activation, Somatic cell nuclear transfer, Animal biotechnology, Genetic engineering

## Abstract

Melanoma is a serious type of skin cancer that originates from melanocytes. Rodent melanoma models have provided valuable insights into melanoma pathology; however, they often lack applicability to humans owing to genetic, anatomical, physiological, and metabolic differences. Herein, we developed a transgenic porcine melanoma model that closely resembles humans via somatic cell nuclear transfer (SCNT). Our model features the conditional oncogenes cassettes, TP53^R167H^ and human BRAF^V600E^, controlled by melanocyte-specific CreER recombinase. After SCNT, transgenic embryos developed normally, with the capacity to develop porcine embryonic stem cells. Seven transgenic piglets with oncogene cassettes were born through embryo transfer. We demonstrated that Cre recombination-mediated oncogene activation remarkably triggered the mitogen-activated protein kinase pathway in vitro. Notably, intradermal injection of 4-hydroxytamoxifen activated oncogene cassettes in vivo, resulting in melanocytic lesions resembling hyperpigmented nevi with increased proliferative properties similar to early human melanomas. This melanoma-inducing system, heritably transmitted to offspring, supports large-scale studies. The novel porcine model provides a valuable tool for elucidating melanoma development and metastasis mechanism, advancing translational medicine, and facilitating preclinical evaluation of new anticancer drugs.

**Supplementary Information:**

The online version contains supplementary material available at 10.1038/s41598-024-82554-w.

## Introduction

Malignant melanoma, which originates from the neoplastic transformation of melanocytes, is highly metastatic and aggressive. It accounts for 75% of all skin cancer-related deaths^1^. Despite the advancements in understanding melanoma biology and improved therapies targeting mutant driver genes and immune checkpoints, its treatment remains challenging owing to its low reactivity and unpredictable resistance^2,3^. Thus, understanding the molecular mechanisms of melanoma initiation and progression is important for innovative therapy development. One of the most frequent genetic alterations observed in human melanoma is the substitution of valine with glutamic acid at position 600 of the BRAF protein (BRAF^V600E^), found in approximately 60% of cases^4^. It activates the downstream mitogen-activated protein kinase (MAPK) pathway^5^. The BRAF^V600E^ mutation accelerates the development of benign melanocytic hyperplasia. In addition to this, disruption of tumor suppressor genes leads to the development of malignant melanoma^6,7^. Delineating the mechanisms underlying these genetic alterations observed in human melanoma patients is crucial for successful melanoma therapies.

Genetically engineered mouse models (GEMMs) are practical tools for modeling human cancer, and several studies have been conducted to identify the genetic changes related to *BRAF*-mutated melanoma using GEMMs^8^. Silencing tumor suppressor genes, such as *PTEN*, *CDKN2A*, or *TP53*, and introducing the *BRAF* mutation results in the development of melanoma in these models, offering valuable insights^7,9–11^. Although considerable advancements have been made in melanoma therapeutic strategies using GEMMs, they have substantial limitations as disease models owing to physical differences compared with humans, such as size and lifespan^12^. Notably, mouse and human skins have distinct anatomical and functional characteristics, including skin thickness, composition, and location of melanocytes, which may affect the initiation and progression of melanoma^8^. For these reasons, using GEMMs as preclinical models for melanoma therapy poses difficulties. Thus, new melanoma animal models that can replace GEMMs and be used in translational research are required.

Pigs are a valuable alternative due to their similarity to humans in size, lifespan, metabolism, anatomy, and physiology^13,14^. The porcine genome is similar in size and composition to the human genome, with a high degree of homology^15–17^. These similarities provide advantages for the establishment of genetically engineered porcine cancer models that can mimic human disease progression and facilitate the evaluation of anticancer drugs^18^. Porcine skin is widely used as a substitute for human skin in dermatological research owing to its anatomical and physiological similarities^19–21^. These include epidermal thickness, dermal-to-epidermal layer ratio, hair follicle content, collagen and lipid composition, pigmentation, and adnexal structures^22,23^. Previously, Sinclair miniature swine and melanoma-bearing Leviekov minipigs (MeLiM), which develop spontaneous metastatic melanoma, were used as porcine models for melanoma research, enabling the investigation of the biological processes underlying melanoma progression and spontaneous regression^24,25^. However, the genetic causes of melanoma in Sinclair and MeLiM remain poorly understood, and the spontaneous occurrence of melanoma makes it difficult to study the early development of melanoma and its location-specific development. Therefore, genetic engineering and induction in a time- or tissue-specific manner are critical for understanding the development and progression of melanomas.

The Cre-*lox*P system is a widely used genetic engineering technology that enables temporal and spatial gene editing through simple manipulation^26,27^. Cre recombinase recognizes and catalyzes the recombination between two *loxP* sites^26,27^. The activation of the inducible Cre system in a time- and cell-specific manner is regulated using cell-specific regulatory elements and inducers, such as tamoxifen^26^. In the tamoxifen-inducible Cre system, the Cre protein is fused to the modified estrogen receptor (ER) with a ligand-binding domain mutation called CreERT^26^. The fused Cre protein typically binds to heat shock protein 90 (HSP90) in the cytoplasm. Upon binding to tamoxifen or 4-hydroxytamoxifen (4-OHT), this interaction is disrupted, and CreERT translocates into the nucleus^26^. To improve efficiency, CreER^T2^, which has ten-fold more sensitivity to tamoxifen or 4-OHT, was generated^26^. Genetically engineered porcine models containing Cre-inducible transgenes have been developed for investigating tumor progression and drug responses in various cancer types, including soft tissue sarcoma, pancreatic ductal adenocarcinoma, and intestinal cancer^28–30^.

In the present study, we present a novel porcine model designed to induce melanoma driven by a controllable melanocyte-specific oncogene expression. Using somatic cell nuclear transfer (SCNT), we generated a transgenic porcine model harboring an oncogene cassette regulated by CreER^T2^ recombinase under the control of the melanocyte-specific tyrosinase (TYR) promoter sequence. Our model demonstrates the ability to effectively activate the oncogenic signaling pathway and facilitate the formation of early melanoma via CreER^T2^ mediated recombination.

## Results

### Construction of the melanoma-inducing system in a porcine model

To develop a porcine melanocyte-specific expression system, the gene regulatory sequences of porcine *TYR* gene, a representative marker of terminally differentiated melanocytes, were discovered based on the functionally defined enhancer and promoter regions of the murine *Tyr* gene (Supplementary Fig. 1). The obtained porcine *TYR* enhancer and promoter (p*TYR*en/pr) sequence showed significantly higher transcriptional activity in human melanocytic cancer, SK-MEL-2, and mouse B16F10 cells than in other types of cells^31^ (Supplementary Fig. 1).

**Fig. 1:**
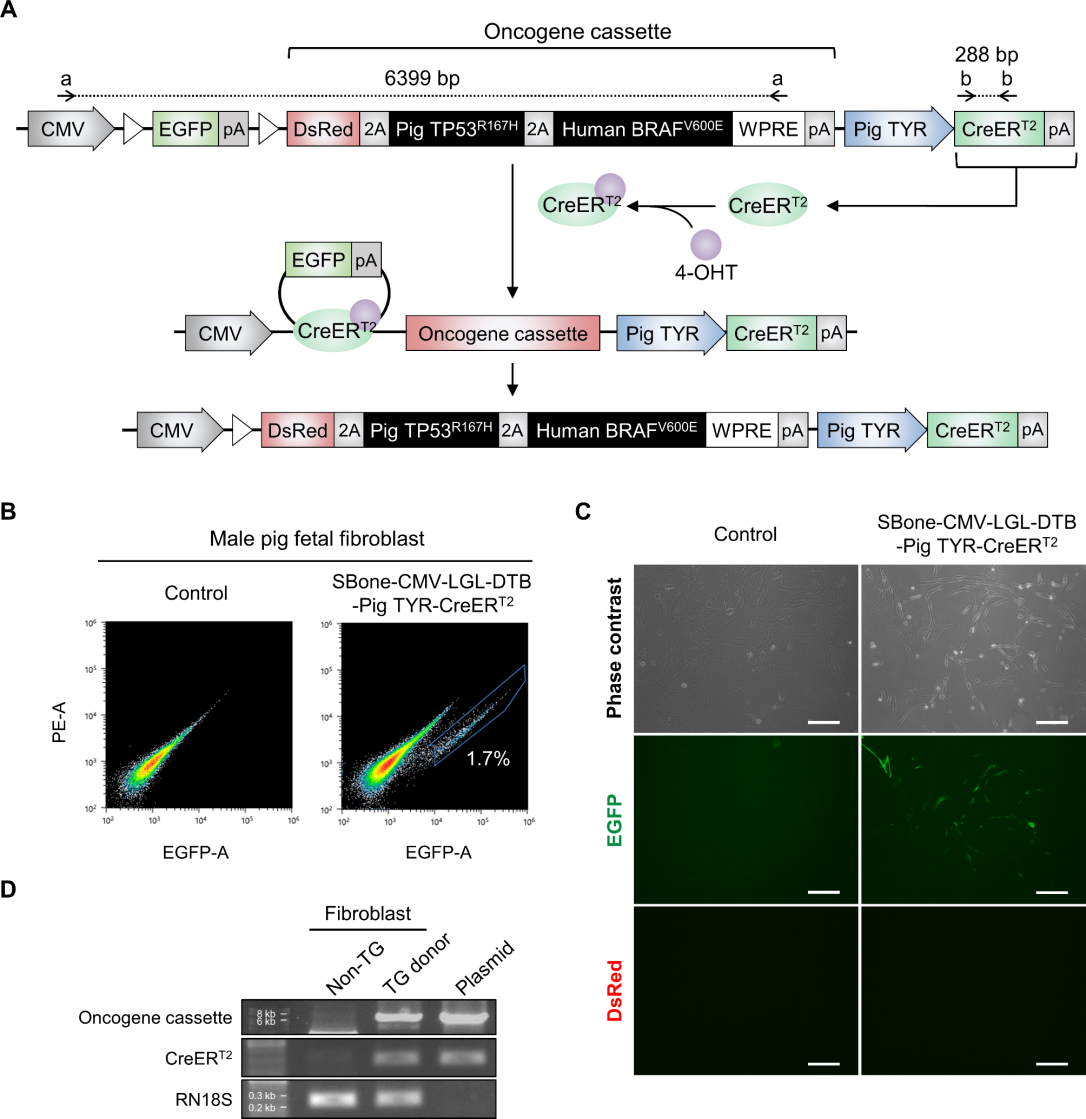
Establishing transgenic donor pig fetal fibroblasts carrying the CreER^T2^ inducible system for SCNT. (**A**) Schematic diagram of the TB-pTYR::Cre system for porcine melanoma model production. The cassette regulates melanocyte-specific expression of CreER^T2^, further inducing recombination of the construct and expression of mutant pig *TP53* and human *BRAF*. Arrows labeled with lower case letters represent primers for PCR analysis to confirm genome integration of the system (a: Oncogene cassette; b: CreER^T2^) (**B**) FACS analysis of the GFP-positive population of transgenic donor fibroblasts carrying the CreER^T2^ inducible system. (**C**) Representative image of GFP or DsRed expression in transgenic donor fibroblasts. Scale bar, 200 μm. (**D**) PCR analysis confirming genomic integration of the CreER^T2^ inducible system in transgenic donor fibroblasts using genomic DNA derived from control and transgenic fibroblasts. Non-transgenic (TG), genomic DNA extracted from normal porcine cell; TG Donor, genomic DNA from the transgenic donor cells; Plasmid, TB-pTYR::Cre plasmid vector.

Recent studies have identified various genetic alterations observed in human melanoma patients using next-generation sequencing^4,32^. Activated human *BRAF*^V600E^, the most frequent mutation in human melanomas, and *TP53*^R167H^, corresponding to the TP53^R175H^ hotspot mutation in human cancers, were used to develop a more clinically relevant porcine model. Gene homology comparison analysis revealed that the human BRAF protein sequence has approximately 97% similarity to that of porcine BRAF (Supplementary Fig. 2). In addition, human BRAF^V600E^ was able to activate the downstream extracellular signal-regulated kinase (ERK)1/2 pathway, affecting the cellular morphology of porcine fibroblasts (Supplementary Fig. 2). Based on these results, a CreER^T2^-*lox*P-based conditional mutant pig *TP53* and human *BRAF* gene expression cassette, named CMV-LGL-DTB-p*TYR*en/pr-CreER^T2^ (hereafter referred to as TB-pTYR::Cre), was developed (Fig. 1A). Briefly, before 4-OHT administration, the TB-pTYR::Cre system expresses only the EGFP gene and terminates transcription at the poly-A signal, preventing oncogene expression in normal status. Upon administration of 4-OHT, it binds to the ER domain of CreER^T2^ expressed by the melanocyte-specific *TYR* promotor sequence, allowing 4-OHT-CreER^T2^ complex to translocate into the nucleus only in melanocytes. Then, Cre recombinase can catalyze the recombination of two *lox*P sites, resulting in the excision of the EGFP-pA sequence and expression of the melanoma-inducing oncogenes.

To establish transgenic donor cells for SCNT, we introduced the TB-pTYR::Cre cassette into porcine fetal fibroblasts using a Sleeping Beauty (SB) transposon system. After serial passaging, we detected fibroblasts expressing enhanced green fluorescent protein (EGFP), which were subsequently sorted using a fluorescence-activated cell sorter (FACS) (Fig. 1B). These fibroblasts stably expressed EGFP but not DsRed (Fig. 1B,C). Integration of the whole TB-pTYR::Cre cassette was confirmed via polymerase chain reaction (PCR) analysis using genomic DNA extracted from the transgenic fibroblasts (Fig. 1D). Thus, we successfully established TB-pTYR::Cre transgenic donor cells for SCNT.

### Confirmation of pluripotent stem cell potential of TB-pTYR::Cre SCNT embryos

**Fig. 2:**
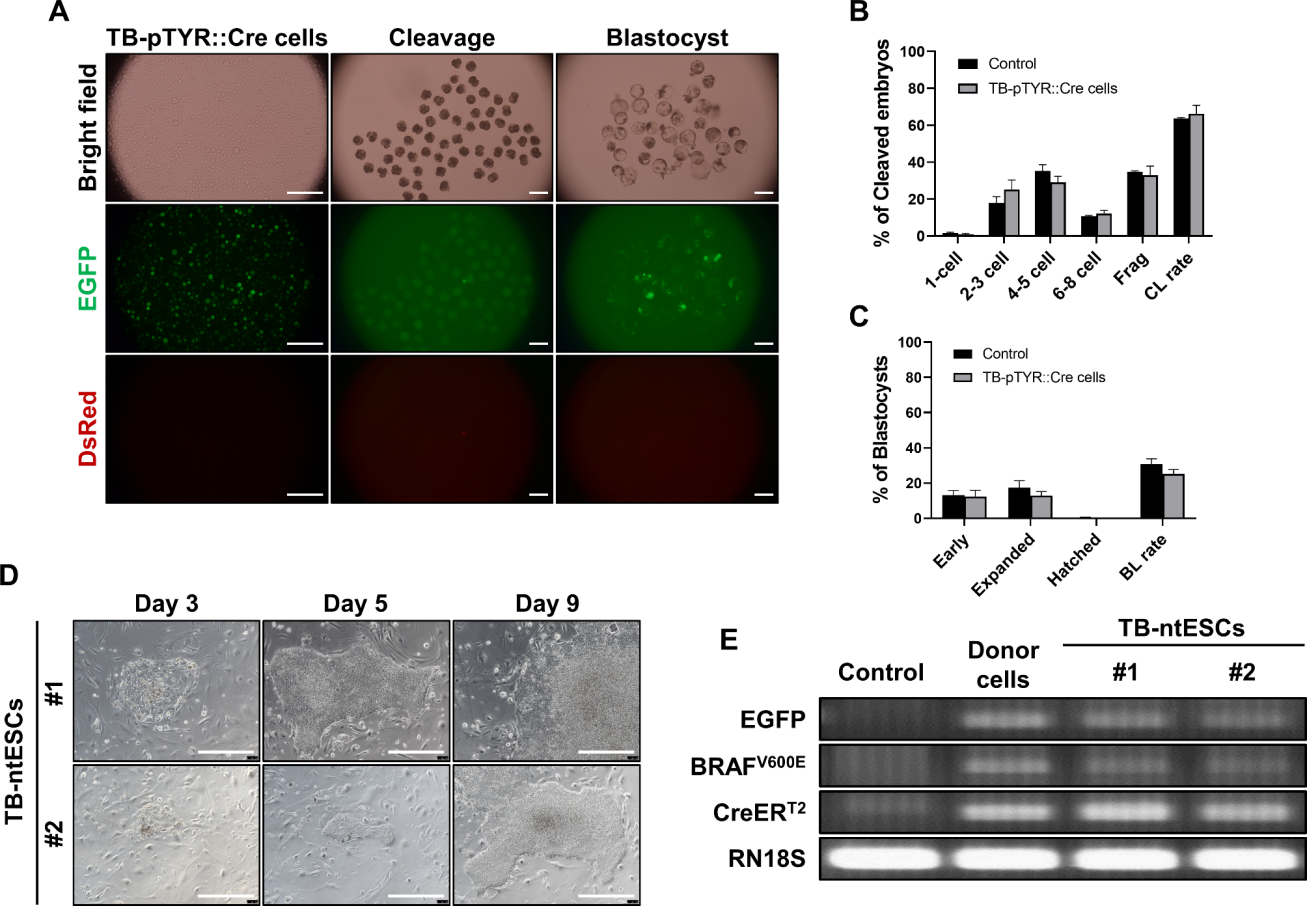
Developmental efficiency of SCNT embryos derived from male TB-pTYR::Cre donor cells. (**A**) Representative images of EGFP-expressing donor cells and SCNT embryos during IVC. Scale bar, 200 μm. (**B**,**C**) Effect of different donor cell types during IVC on the cleavage (**B**) and blastocyst (**C**) formation pattern of SCNT embryos. The cleavage rate was measured on day 2, while the blastocyst formation rate was evaluated on day 7 of culture. The experiment was performed in triplicate. Data are presented as mean ± SEM, n = 3. The data were analyzed using Student’s t-test. Control, normal pig fetal fibroblasts; Frag, fragmentation; CL, cleavage; BL, blastocyst. (**D**) The outgrowth morphologies of two TB-ntESC lines established from SCNT-derived blastocysts. Scale bar, 100 μm. (**E**) Transgene identification in genomic DNA from two TB-ntESC lines via PCR. *RN18S* was used as a control.

Since BRAF^V600E^ expression causes embryonic lethality^33^, we initially assessed the in vitro development after SCNT to confirm whether TB-pTYR::Cre donor cells undergo normal embryonic development. Cleavage and blastocyst morphology were observed on days 2 and 7 after SCNT, respectively. EGFP expression was observed at all stages, whereas DsRed expression was not detected (Fig. 2A). We subsequently examined the cleavage and blastocyst formation patterns of control and TB-pTYR::Cre donor cells and found no significant differences (Fig. 2B,C; Table 1). These results indicated that the oncogene cassette within TB-pTYR::Cre donor cells did not affect embryonic development following SCNT.

**Table 1:** Effect of different donor cell types during IVC on embryonic development after SCNT.

Cell line	No. of oocytes	Fused (%)^†^	No. (%)* of embryos developed to	
			≥ 2-cell	Blastocyst
Control	369	322 (87.2 ± 1.9)	205 (63.6 ± 0.6)	98 (30.9 ± 2.9)
TB-pTYR::Cre cells	281	242 (85.9 ± 2.0)	159 (66.2 ± 4.6)	62 (25.2 ± 2.6)

The experiment was performed in triplicate. All data were presented as mean ± SEM.

*Percentage of total fused oocytes.

^†^Percentage of total oocytes.

Pluripotent stem cells that mimic specific embryonic stages in vitro are extensively used as models for studying early mammalian development^34^. Notably, establishing embryonic stem cells (ESCs) from SCNT-derived blastocysts requires blastocysts of sufficient quality^35,36^. Hence, we confirmed that SCNT blastocysts produced using TB-pTYR::Cre donor cells were of sufficient quality for ESC derivation and assessed the pluripotency and developmental potential of the established ESCs. On day 3, after seeding, seven SCNT blastocysts had attached, resulting in two outgrowths. After further passaging, stable colonies of porcine ESCs with dome-shaped cells were established (termed TB-pTYR::Cre-nuclear transfer ESCs; TB-ntESCs) (Fig. 2D and Supplementary Fig. 3). PCR analysis confirmed that both TB-ntESCs carried an oncogene cassette identical to that of donor cells (Fig. 2E). We observed that TB-ntESCs exhibited normal positive AP staining, diploid karyotypes, and expression of pluripotent stem cell markers OCT4, SOX2, SSEA4, and GFP (Supplementary Fig. 3). Specifically, using a spontaneous embryoid body (EB) differentiation assay, we found that TB-ntESCs expressed EGFP but not DsRed on day 7 and differentiated into the three germ layers (Supplementary Fig. 3). These results demonstrated that SCNT blastocysts using TB-pTYR::Cre donor cells are adequately qualified for ESC generation. Moreover, the resulting TB-ntESCs displayed normal pluripotency and developmental potential.

### Generation of TB-pTYR::Cre transgenic pigs

**Fig. 3:**
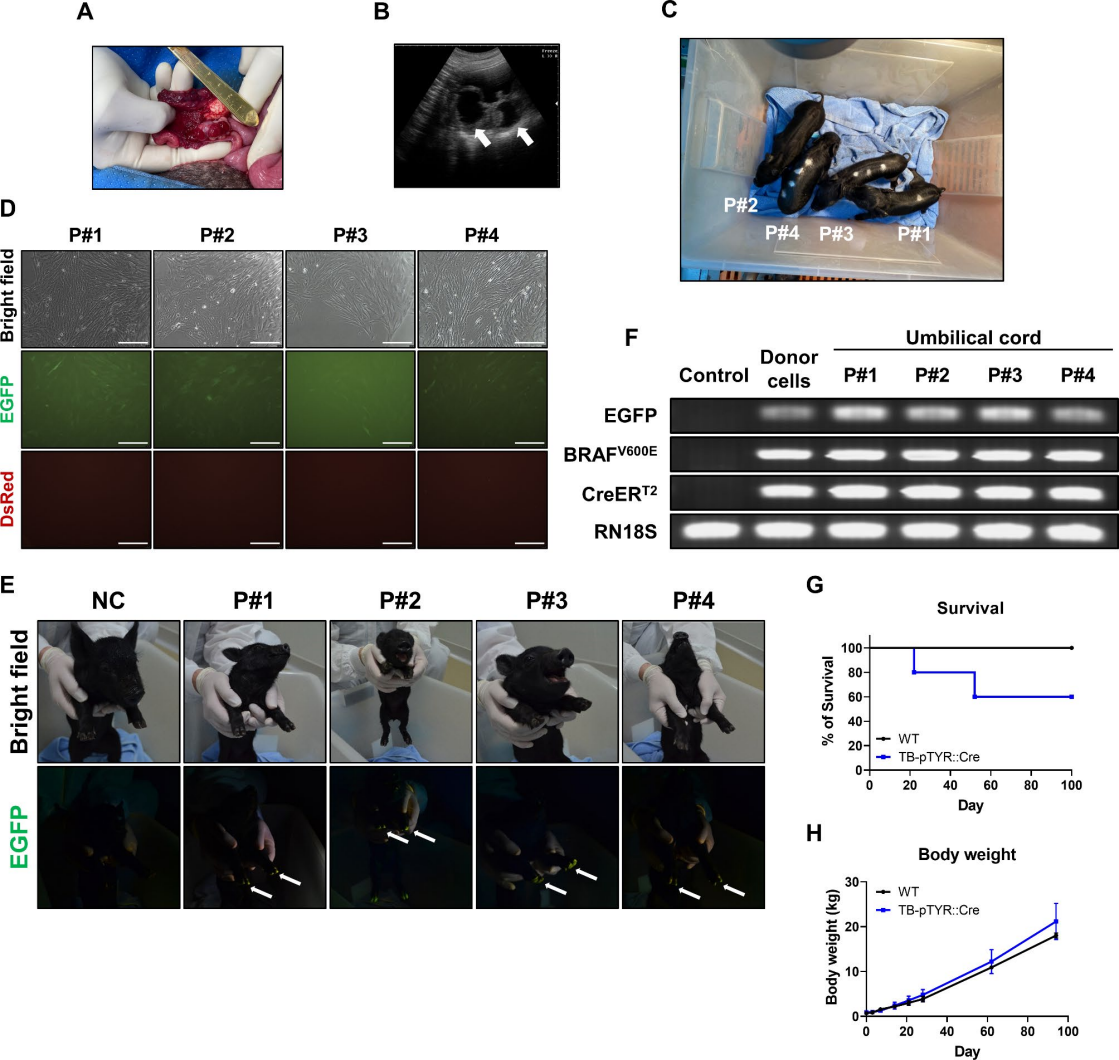
Production of transgenic piglets with TB-pTYR::Cre transgene constructs. (**A**) Image of surrogate F11-58’s ovary during embryo transfer. (**B**) Picture of pregnancy diagnosis from surrogate F11-58 on day 28 after embryo transfer. Bold arrow, embryonic sac. (**C**) Image of TB-pTYR::Cre piglets. P, piglet. (**D**) Representative images showing EGFP expression in umbilical cord cells from each TB-pTYR::Cre piglet. Scale bar, 300 μm. (**E**) Images show EGFP expression detected in hooves under ultraviolet light in each TB-pTYR::Cre piglet. Thin arrow, EGFP expression. NC, negative control. (**F**) Transgene identification in genomic from the umbilical cord of TB-pTYR::Cre piglets via PCR analysis. Control, normal pig fetal fibroblasts. *RN18S* was used as a control. (**G**) Survival curve of TB-pTYR::Cre pigs (n = 5 for each group). (**H**) Body weight of TB-pTYR::Cre pigs (n = 3 for each group). Data are presented as mean ± SEM. The data were analyzed using Student’s t-test.

Embryos (*n* = 356) were cloned using SCNT and transferred into two surrogate pigs during estrus to generate TB-pTYR::Cre pigs (Table 2; Fig. 3A, and Supplementary Fig. 4). Two pregnancies were diagnosed on day 28 using ultrasonography and were developed to full term (Fig. 3B and Supplementary Fig. 4). Six live-born (P#1–5, and P#7) and one stillborn (P#6) piglet were obtained from the two pregnancies (Fig. 3C and Supplementary Fig. 4). EGFP expression was detected in the umbilical cord cells and hooves of each TB-pTYR::Cre piglet (Fig. 3D,E and Supplementary Fig. 4). In addition, the oncogene cassette was detected in all pigs, consistent with the TB-ntESC results (Fig. 3F and Supplementary Fig. 4).

**Table 2:** Summary of embryo transfer results.

Surrogate ID	Ovulation status	No. of transferred embryos	Pregnancy diagnosis	Parturition				
				No. of piglets born (alive/total)	No. (%) of transgenic piglets	No. (%) of offspring		Weight of piglets (g)*
						Abnormal	Live until weaning	
F11-58	J.B.O.	172	Positive	4/4	4 (100)	1 (25)	3 (75)	910 ± 43
GY-18	J.B.O.	184	Positive	2/3	3 (100)	1 (33.3)	1 (33.3)	443.3 ± 20.3

*J.B.O.* just before ovulation.

*The weight of piglets is expressed as the mean ± SEM.

Unexpectedly, TB-pTYR::Cre P#2 was born with unintended tumors with densely pigmented cells which also spread into visceral organs (Supplementary Fig. 5). Melanoma induced in TB-pTYR::Cre P#2 showed significantly increased proliferation and melanoma-related cellular pathways (Supplementary Fig. 5). In addition to the TB-pTYR::Cre P#2 and one stillborn piglet, TB-pTYR::Cre P#5 and P#7 died at 52 and 22 d, respectively (Fig. 3G). These pigs did not develop tumors, indicating early mortality likely due to SCNT. The three remaining TB-pTYR::Cre pigs (P#1, 3, and 4) maintained normal body weights compared to wildtype (WT) pigs and did not exhibit any noticeable phenotype during the observation period preceding 4-OHT induction (Fig. 3H). To investigate genomic characteristics of TB-pTYR::Cre pigs (P#1–4), we initially analyzed a copy number of oncogene cassettes in each transgenic pig (Supplementary Fig. 6). Quantitative PCR analysis of their genomic DNA samples confirmed that P#1, P#3, and P#4 had a 1.5-fold increase in TP53 gene copies compared to a normal pig, which has two endogenous TP53 gene sequences. This result indicates that they carried a single copy of the oncogene cassette. On the other hand, P#2, which showed a 3.5-fold increase, was found to carry five copies of the transgene cassette. This may be associated with unintended tumor formation due to the unpredictable copy number and uncontrollable overexpression of the transgene^37^. Furthermore, the oncogene cassette insertion was validated between the *TBCID2* and *GABBR2* genes on chromosome 1 in P#3, while the cassette was located between the *TNS1* and *RUFY4* genes on chromosome 15 in P#4. Importantly, it has been confirmed that the integrated cassettes in P#3 and P#4 pigs did not disrupt any endogenous gene sequences. However, in P#1, in addition to the correct insertion of the full cassette sequence, the integration of an unintended partial fragment of the cassette involving a sequence that extends beyond the inverted repeat sequence of the transposon element was also detected. Thus, P#3 was considered as a parental generation for our TB-pTYR::Cre pig models to produce their transgenic offspring, while P#1 and P#4 were used to validate TB-pTYR::Cre melanoma induction system in vivo.

### In vitro validation of the oncogene cassette system in the TB-pTYR::Cre pig model

**Fig. 4:**
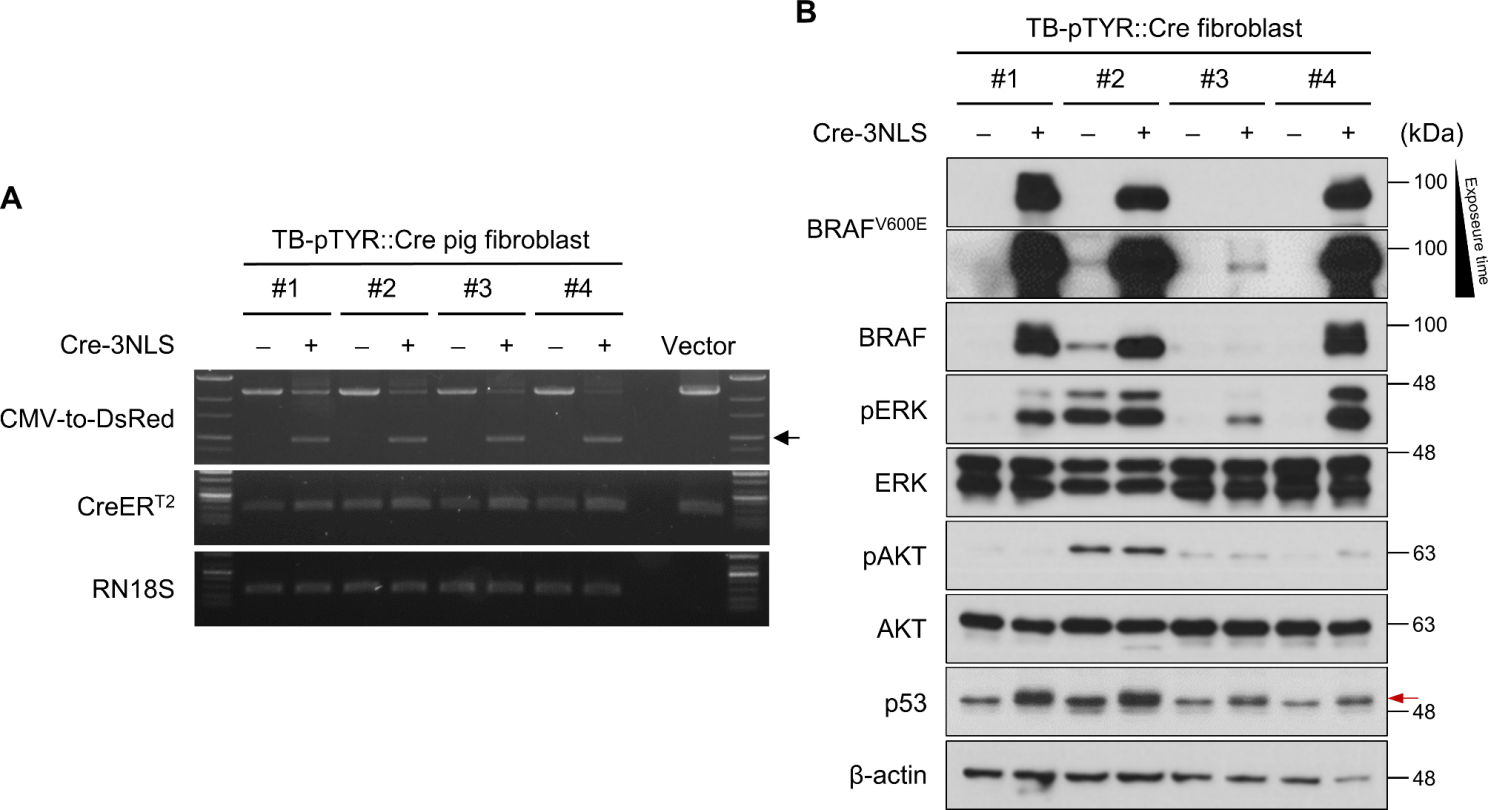
In vitro validation of the Cre-*lox*P system in TB-pTYR::Cre pigs. (**A**) PCR analysis of genomic DNA of TB-pTYR::Cre pig fibroblasts after Cre-3NLS introduction. The arrows indicate shortened PCR amplificons resulting from Cre recombination and the excision of the EGFP-pA sequence. (**B**) Western blot detection of BRAF^V600E^, phosphorylated ERK1/2, phosphorylated AKT, and p53^R167H^ in control and Cre-3NLS-introduced TB-pTYR::Cre pig fibroblasts. Red arrows indicate p53^R167H^ expressed from the oncogene cassettes, which are slightly larger than wildtype p53 due to the remaining 2A peptides.

To validate the efficacy of inducible recombination of the genome in TB-pTYR::Cre pigs, we introduced Cre with a nuclear localization signal (Cre-3NLS) into fibroblasts from TB-pTYR::Cre P#1–4 via electroporation. We performed PCR analysis on genomic DNA extracted from fibroblasts transfected with either control or Cre-3NLS to confirm genomic recombination. Consequently, exogenous Cre-3NLS induced recombination of two *lox*P sites, resulted in shorter amplificons due to excision of *EGFP*-pA sequence (Fig. 4A). Following genomic recombination, Cre-3NLS-introduced fibroblasts exhibited induced expression of human BRAF^V600E^ (Fig. 4B). The expression of human BRAF^V600E^ increased the ERK1/2 phosphorylation in the fibroblasts of TB-pTYR::Cre pigs (Fig. 4B). As it is known that BRAF activates MAPK signaling cascade involving phosphorylation of MEK1/2-EKR1/2, but not PDK-AKT pathway^38,39^, AKT activation was not observed. In addition to the expression of mutant BRAF, exogenous mutant p53 which showed a slightly larger size due to the remaining 2A peptide sequence was also detected upon the recombination (Fig. 4B). These results indicated that our TB-pTYR::Cre system was sufficient for inducing oncogene expression and activating its downstream signaling depending on Cre-mediated recombination in vitro.

### In vivo validation of the oncogene cassette system in the TB-pTYR::Cre pig model

**Fig. 5:**
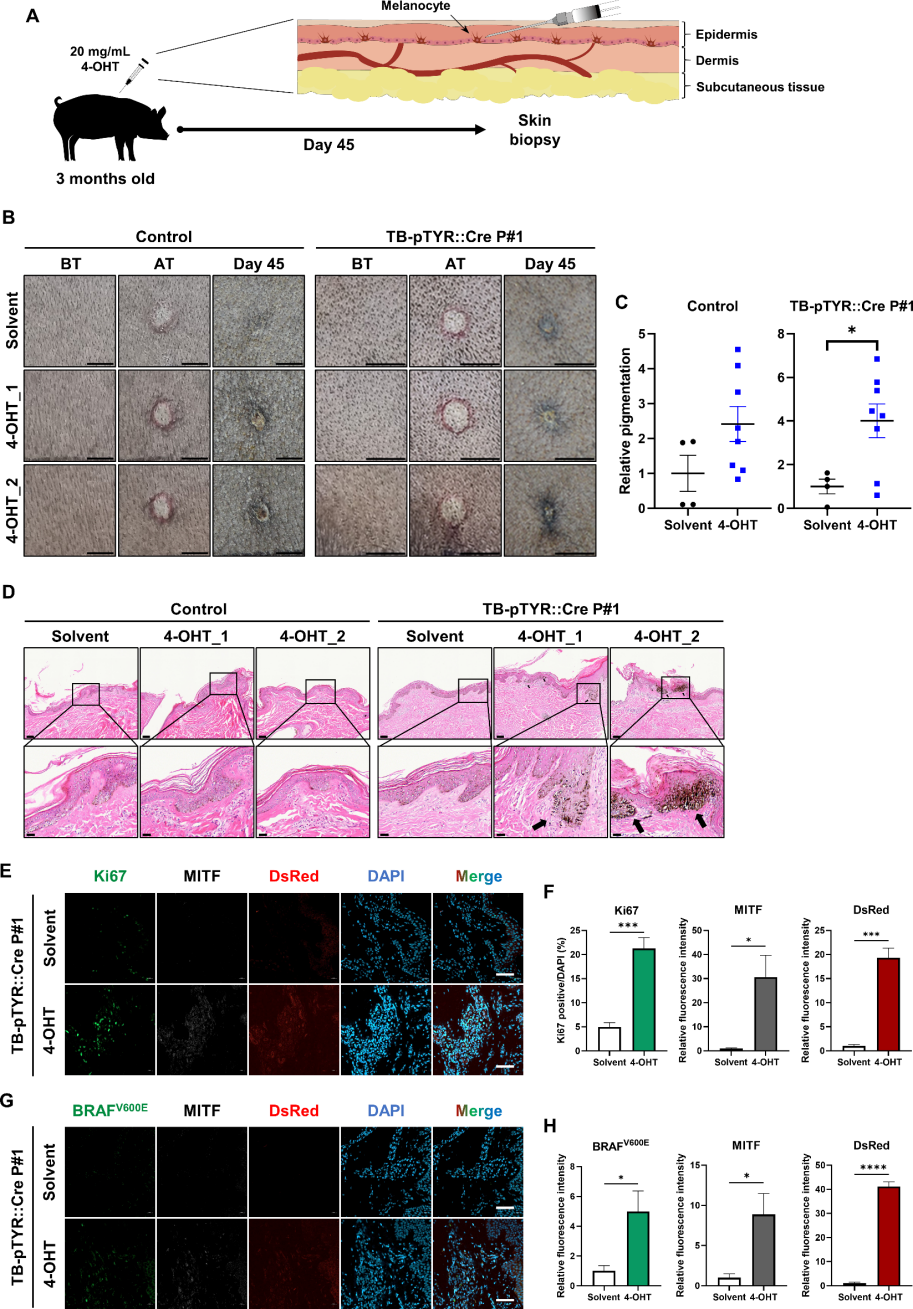
In vivo validation of the TB-pTYR::Cre construct system after 4-OHT treatment. (**A**) Scheme of 4-OHT administration. The skin of an age-matched pig (Control or TB-pTYR::Cre P#1) was shaved, followed by an intradermal injection of 4-OHT. (**B**) Representative images of skin treated with solvent or 4-OHT via intradermal injection. BT, before treatment. AT, after treatment. Scale bar, 1 cm (**C**) Quantification of pigmentation after 45 days in sites treated with solvent or 4-OHT via intradermal injection. Data are presented as mean ± SEM. The data were analyzed using Student’s t-test. *p < 0.05. (**D**) Representative skin histology using H&E staining 45 d post-4-OHT treatment. Black arrow, melanocytic hyperplasia. Scale bar, 100 μm (up) and 30 μm (down). (**E**) Representative immunofluorescence image of Ki67, MITF, and DsRed expression in TB-pTYR::Cre P#1 skin following solvent and 4-OHT treatment. Scale bar, 150 μm. (**F**) Quantification of Ki67, MITF, and DsRed expression in TB-pTYR::Cre P#1 skin following solvent and 4-OHT treatment. Data are presented as mean ± SEM, n = 4. The data were analyzed using Student’s t-test. *p < 0.05, ***p < 0.001. (**G**) Representative immunofluorescence image of BRAF^V600E^, MITF, and DsRed expression in TB-pTYR::Cre P#1 skin following solvent and 4-OHT treatment. Scale bar, 150 μm. (**H**) Quantification of BRAF^V600E^, MITF, and DsRed expression in TB-pTYR::Cre P#1 skin following solvent and 4-OHT treatment. Data are presented as mean ± SEM, n = 4. The data were analyzed using Student’s t-test. *p < 0.05, ****p < 0.0001.

To demonstrate the feasibility of activating the TB-pTYR::Cre cassette in vivo, P#1 was used for an in vivo validation of our melanoma-inducing system. We administered a treatment of 20 mg/mL 4-OHT via intradermal injection, facilitating direct exposure of melanocytes to 4-OHT (Fig. 5A). TB-pTYR::Cre P#1 and a control pig exhibited bleb formation immediately after intradermal injection of 4-OHT and solvents. After 45 d after 4-OHT treatment, TB-pTYR::Cre P#1 exhibited significantly elevated pigmentation in the 4-OHT-treated sites compared with solvent-treated sites, although no difference was observed in a control pig (Fig. 5B,C). In the epidermis of a control pig treated with either the solvent or 4-OHT, we observed focal crust formation and mild orthokeratotic hyperkeratosis consisting of neutrophils, plasma components, and keratinocytes. In contrast, in TB-pTYR::Cre P#1, the solvent-treated sites exhibited the same symptoms as those in a control pig; however, marked melanocytic hyperplasia at the dermoepidermal junction was observed in the 4-OHT-treated sites (Fig. 5D).

To investigate the histological characteristics of pigmented skin tissue induced by 4-OHT in TB-pTYR::Cre P#1, we performed immunofluorescence staining for Ki67, human BRAF^V600E^, microphthalmia-associated transcription factor (MITF), GFP and DsRed. Compared with solvent-treated skin, 4-OHT treatment decreased GFP expression and significantly increased the expression of DsRed, indicating Cre-dependent recombination, as well as that of MITF, indicating increased pigmentation (Fig. 5E,F, and Supplementary Fig. 7). In addition, 4-OHT-treated skin was characterized by a significantly increased population of Ki67-positive cells, indicating a higher proliferation rate, potentially attributed to oncogenic transformation (Fig. 5E,F). Notably, 4-OHT treatment resulted in significantly higher expression of human BRAF^V600E^, indicating the oncogene cassette expression following Cre-dependent recombination (Fig. 5G,H). Collectively, these results indicated that the TB-pTYR::Cre pig model can induce oncogene cassette expression in vivo via the intradermal injection of 4-OHT, leading to nevus-like formation characterized by melanocytic hyperplasia and increased proliferation.

### Germline transmission of the oncogene cassette system in the TB-pTYR::Cre pig model

A normal reproductive capacity of animal models, which enables them to produce their offspring with identical genetic characteristics, is crucial for ensuring the study of cancer phenotypes and the effective validation of preclinical models^14,40^. Therefore, we assessed the fertility of TB-pTYR::Cre pigs and examined the germline transmission potential of TB-pTYR::Cre cassettes. EGFP expression was observed in the semen of male TB-pTYR::Cre P#3 after sexual maturation (Supplementary Fig. 8). In vitro fertilization (IVF) was subsequently performed using semen from TB-pTYR::Cre P#3, with IVF embryos developing normally to the cleavage (56.1 ± 3.6%) and blastocyst (11.5 ± 2.9%) stages (Supplementary Fig. 8). PCR analysis of genomic DNA from TB-pTYR::Cre P#3 sperm and a single blastocyst produced via IVF revealed the presence of a *CreER*^*T*2^ insertion (Supplementary Fig. 8). Furthermore, immunostaining confirmed GFP expression in the cytoplasm of the cleavages and blastocysts generated using TB-pTYR::Cre P#3 sperm, consistent with the TB-ntESC results (Supplementary Fig. 8).

**Fig. 6:**
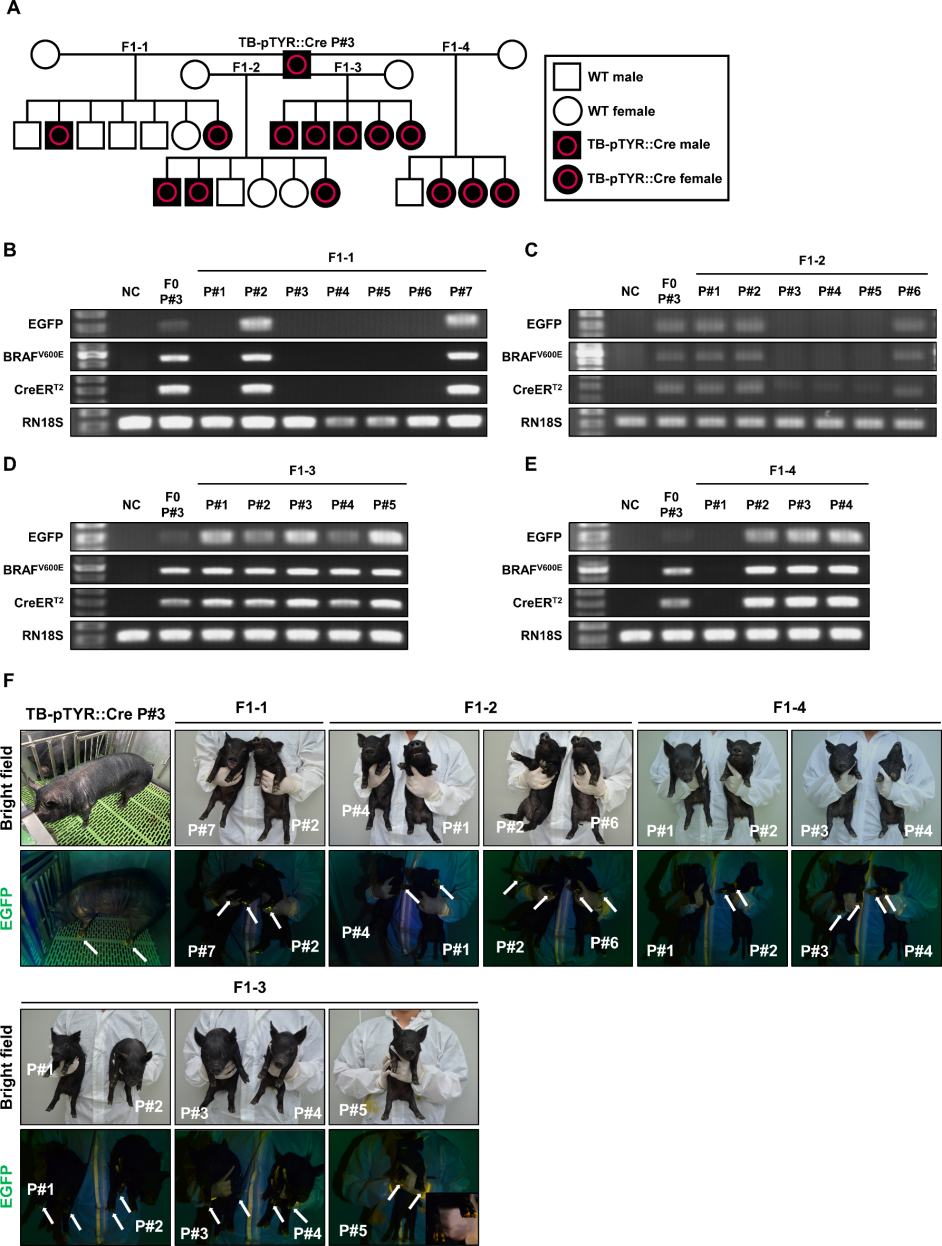
TB-pTYR::Cre pig generation via natural mating. (**A**) Pedigree of a cross between a founder (F0) TB-pTYR::Cre P#3 pig and four WT pigs. (**B**–**E**) Transgene identification in genomic DNA isolated from F1-1, F1-2, F1-3, and F1-4 generation using PCR analysis. NC, negative control. P, piglet. *RN18S* was used as a control. (**F**) Images of EGFP expression detected in hooved under ultraviolet light in each F1 TB-pTYR::Cre piglet. Thin arrow, EGFP expression.

The male founder (F0 TB-pTYR::Cre P#3) pig was mated with WT female Jeju native pigs to generate F1 TB-pTYR::Cre pigs (Fig. 6A). PCR analysis showed that of the 22 F1 offspring pigs, 13 carried the oncogene cassette (Fig. 6B–E). Similar to the male founder, the hooves of each of these 13 F1 TB-pTYR::Cre piglets exhibited EGFP expression, whereas F1 pigs without the oncogene cassette did not exhibit EGFP expression (Fig. 6F). The sex ratio in F1 TB-pTYR::Cre pigs was 46.2% males and 53.8% females (Table 3). These data suggest that the TB-pTYR::Cre oncogene cassette present in TB-pTYR::Cre pigs is capable of germline transmission both in vitro and in vivo.

**Table 3:** Summary of the production of F1 TB-pTYR::Cre piglets.

Generation	TB-pTYR::Cre piglets	WT
F1-1	♂: 1, ♀: 1	♂: 4, ♀: 1
F1-2	♂: 2, ♀: 1	♂: 1, ♀: 2
F1-3	♂: 3, ♀: 2	–
F1-4	♀: 3	♂: 1
Total	♂: 6 (46.2%) ♀: 7 (53.8%)	♂: 6 (66.7%) ♀: 3 (33.3%)

## Discussion

Several spontaneous melanoma porcine models exist, such as the MeLiM, Munich miniature, Troll, and Sinclair miniature swine. Although these models develop melanoma similar to that in humans, their uneven spontaneous development of tumors makes research challenging. Herein, we developed a porcine melanoma model using our temporally and spatially controllable melanocyte-specific oncogene expression system based on CreER-*lox*P. This model allows for melanoma development through the expression of TP53^R167H^ and BRAF^V600E^, which are mutations commonly found in melanomas. Upon Cre activation in vitro and in vivo, our TB-pTYR::Cre pigs exhibited activation of oncogenic signaling pathways and melanoma induction. Furthermore, our model demonstrated reproductive capacity, suggesting that TB-pTYR::Cre pigs are a practical model for investigating the biological and molecular characteristics of melanoma.

A tissue- or cell-type-specific gene regulation system is crucial for achieving successful recombination exclusively in target cells using the CreER-*lox*P system^41^. *TYR*, a gene encoding a key enzyme involved in melanin synthesis, is a marker gene of terminally differentiated melanocytic cells^42^. To explore the functional gene regulatory sequences of the porcine *TYR* gene, we analyzed its potential promoter region by a comparative analysis using functionally validated enhancer and promoter regions of the murine *Tyr* gene. Subsequently, we successfully established and validated a melanocyte-specific expression system using conserved enhancer and promoter sequences of the porcine *TYR* gene. Unlike previous onco-pig models that induced tumor driver gene expression in a non-cell type-specific manner via local adenovirus delivery^28–30^, our *TYR* enhancer/promoter-based model enables the control of oncogene expression, specifically in melanocytes. Therefore, our model is more suitable and promising for melanoma initiation and development.

We confirmed that the oncogenes in our system were not expressed under normal conditions, and their expression was detected only after exogenous Cre introduction in vitro. Unexpectedly, one piglet (TB-pTYR::Cre P#2) developed an unexpected tumor showing densely pigmented lesions and cell infiltration to the dermis and subcutaneous tissue, which are typical histological characteristics of human melanoma (Supplementary Fig. 5). Additionally, metastatic lesions were observed in the lungs and liver. The pigmented lesions showed increased expression of *CreER*^*T*2^, *BRAF*^*V600E*^ and activated MAPK pathway, also expressing DsRed. In addition, compared with other piglets, BRAF^V600E^ expression and ERK phosphorylation were elevated before the introduction of Cre with multiple copies of oncogene cassette (Fig. 4B and Supplementary Fig. 6A). These results indicated that the Cre-inducible system was activated independently of 4-OHT in TB-pTYR::Cre P#2. The CreER^T2^-inducible system can be activated and translocated into the nucleus without tamoxifen treatment^43–45^. In addition, the *Tyr::CreER*^*T2*^; *PTEN*^*−/−*^; *BRAF*^*V600E/+*^ cassette was reported to result in spontaneous melanoma in GEMM mice due to leakage of the inducible Cre system^46^. Such leakage may occur when the inducible Cre system is expressed under a strong promoter^47^. Additionally, cleavage of Cre and the ligand-binding domain by intracellular proteases may cause recombination in the absence of tamoxifen^48^. Further research on additional melanocyte-specific promoters or modifying CreER^T2^ could enhance the stability of the Cre-*lox*P system.

Here, we successfully induced early melanotic lesions through intradermal injection of 4-OHT. However, some potential regional effect needs to be addressed. 4-OHT topical administration to ear or tail skin was insufficient to induce melanoma in the TB-pTYR::Cre pig (P#4) (Supplementary Fig. 9). These differences may result from varying skin thicknesses and melanocyte distributions. In mice, melanocytes are primarily located in the bulb region of hair follicles, whereas in pigs, they are distributed in the basal layer of the epidermis and hair follicles^8,21^. It implies that optimization of appropriate intradermal delivery is required so that 4-OHT could reach a region of melanocytes in the porcine skin. Developing 4-OHT delivery via microneedle patches or other transdermal delivery methods optimized for pig models could be an alternative way to activate oncogene cassettes into the skin with less pain and greater efficiency^49^. Additionally, we have tested whether the oral administration of 4-OHT could efficiently induce melanoma in the TB-pTYR::Cre pig (P#4) (Supplementary Fig. 9). However, no visible melanocytic lesion was observed. Previous reports showed that activation of the oncogenic system in organ tissue-specific transgenic pigs was primarily achieved via oral administration of 4-OHT^30,50^. This implies that penetration of orally administrated 4-OHT into skin tissue was also not successful in terms of pharmacokinetics and pharmacodynamics. There could be a couple of potential causes such as insufficient 4-OHT concentration and administration schedule. Since oral administration of 4-OHT is an easily accessible and painless manner to animals, it needs to be further optimized.

Transgenic overexpression of BRAF^V600E^ combined with the loss of Trp53 expression induces malignant melanoma in fish^51^, whereas in mice, mutant Trp53 promotes BRAF mutation-derived melanomagenesis associated with ultraviolet radiation-induced DNA damage^10^. These models exhibited local pigmentation patterns within 2–7 days. Unlike in previous animal models of melanoma, lesion development in our porcine model was not rapid. After induction, we observed nevus-like formation with melanocytic hyperplasia and an increased proliferative index, consistent with the initiation stage of melanoma. The metastatic development of melanoma in humans can take up to 5 years^52^. Given the extended duration of development, we believe that our porcine model more closely resembles the human condition than other animal models. In addition, long-term observations following induction are necessary to study the metastatic development of melanomas. This progression of melanoma development also correlates with the overall mutational burden^53^. A higher burden of BRAF mutations leads to accelerated melanoma development, with copy number alterations being associated with lethal development^7,54^. Given that our porcine model allows germline transmission of the oncogene cassette, the rapid metastatic development of melanomas can be expected in homozygous individuals through breeding.

To the best of our knowledge, this study represents the first report on a melanoma-inducible porcine model driven by a melanocyte-specific inducible Cre-*lox*P system harboring TP53^R167H^ and human BRAF^V600E^. Unlike spontaneous melanoma porcine models, our model has the advantage of temporal and spatial inducibility that enables neoplastic melanocyte measurement. Further, we bring forth a novel approach to assess Cre-*lox*P system activation in porcine skin. The interpretation of our findings, is however, limited at present by several factors, including the low sample size of experimental animals, the single instance of successful systemic induction, and the early-stage identification of melanoma. Further studies that comprise a larger cohort and make observations over a longer duration are thus essential to validate our findings. This porcine model allows investigation of the crucial disparities in melanoma development across different stages due to the longer lifespan of pigs as compared to that of mice. Consequently, our porcine melanoma model may potentially serve as a novel platform for elucidating the biological mechanisms of melanoma, advancing translational research, and enabling the preclinical evaluation of novel anticancer agents.

## Methods

### Animals and ethics declaration

Jeju native pigs^55^ used in this study were maintained at Cronex Inc. (Cheongju, South Korea). All animal experiments were approved by the Committee on Ethics of Animal Experiments at Chungbuk National University (Approval number: CBNUA-1735-22-02). All surgeries and experiments were performed under anesthesia to minimize animal suffering. For preanesthesia, a mixture of ketamine and xylazine in a 1:3 ratio was administered via the ear veins. Respiratory anesthesia was then maintained using isoflurane liquid. For euthanasia, an overdose of succinylcholine (1 mL/10 kg) was administered following anesthesia. Additionally, all methods were conducted in accordance with relevant guidelines and regulations, and in compliance with the ARRIVE guidelines 2.0.

### Cloning of the pig *TYR* promoter

Porcine *TYR* enhancer and promoter sequences were cloned from the genomic DNA of *Sus scrofa* miniature pigs using PCR amplification. Genomic DNA was extracted from Yucatan miniature pig fibroblasts using a Wizard Genomic DNA Extraction Kit (Promega, Madison, WI, USA) according to the manufacturer’s instructions. Thereafter, the enhancer (− 10876 to − 9318 upstream from its transcription start site, TSS) and promoter (− 388 to + 47 from TSS) regions of porcine *TYR* were amplified using the PrimeSTAR® GXL DNA Polymerase (TaKaRa Bio, Inc., Otsu, Shiga, Japan) with the following primer sets: Enhancer (Forward, F) 5′-gctagcgtcaggcatcatctttcccc-3′ (Reverse, R): 5′-ggtacctcagtcttacccccaccaaat-3′; Promoter (F) 5′-ggtaccacaaccatcttgcacccaaa-3′; (R) 5′-tctagatcctctagttctcacaaggtc-3′. Each product was digested with *NheI*-*KpnI* and *KpnI*-*XbaI*. Subsequently, the obtained amplicons were integrated into the pig *GFAP* promoter region of the pcDNA3.1-pig GFAP-CreER^T2^ plasmid vector^56^. The final pcDNA3.1-pig TYR-CreER^T2^ vector was sequenced using an ABI BigDye terminator v3.1 Cycle Sequencing Kit (Applied Biosystems, Foster, CA, USA).

### Semi-quantitative PCR

To compare the expression of endogenous *TYR*, total RNA was extracted using TRIzol reagent (TaKaRa Bio, Inc.) according to the manufacturer’s protocol. The isolated RNA was amplified in a 20 µL PCR reaction using PrimeSTAR® GXL DNA polymerase. PCR conditions were as follows: pre-denaturation at 98 °C for 5 min, 25 cycles of denaturation at 98 °C for 30 s, annealing at 60 °C for 20 s, extension at 68 °C for 1 min/1-kbp, followed by 68 °C for 5 min. The amplified PCR products were analyzed using gel electrophoresis.

### Analyzing *TYR* promoter activity in vitro

To confirm the activity of the cloned *TYR* promoter, the pcDNA3.1-pig TYR-CreER^T2^ vector was transfected with second-generation lentiviral packaging plasmids Δ8.9 and pVSV.G using the LipoJet™ in vitro DNA transfection kit (SignaGen Laboratories, Frederick, MD, USA) in transformed human embryonic kidney (HEK) 293T cells (American Type Culture Collection, Manassas, VA, USA). The culture medium was harvested 24 h after transfection. The lentivirus was filtered through a 0.45 μm syringe filter and concentrated using a Lenti-X™ Concentrator (TaKaRa Bio, Inc.). HEK293T, U87MG, SK-MEL-2, Ink4a/Arf^−/−^ mouse astrocyte, and B16F10 cells were infected with the virus in the presence of 6 µg/mL polybrene (Sigma-Aldrich). Cells were selected in Dulbecco’s modified Eagle’s medium (DMEM, Gibco) supplemented with 10% fetal bovine serum (FBS) and 1 µg/mL puromycin (Invitrogen, Carlsbad, CA, USA) for 7 d. *TYR* promoter activity was analyzed using flow cytometry (FACSVerse, BD Biosciences, Franklin Lakes, NJ, USA).

### Validation of human BRAF^V600E^ in porcine fibroblasts

To validate the functional activity of human BRAF^V600E^ in porcine fibroblasts, pCDH-CMV-BRAF^V600E^-puro was transfected into HEK293T cells to generate a lentivirus. Yucatan and Jeju porcine fibroblasts were infected with the virus in the presence of 6 µg/mL polybrene. Microscopic images of cellular morphology were obtained 3 d after infection.

### Establishment of transposon-mediated oncogene expression system

The SB transposon-mediated pT4/HB-CMV-LoxP-EGFP-LoxP-DsRed-P2A-porcine TP53^R167H^-T2A-human BRAF^V600E^-pig TYR-CreER^T2^ (CMV-LGL-DTB-pTYR-CreER^T2^) was constructed. Briefly, the CMV-G^Cas9^-DSH plasmid vector was used to establish the CreER^T2^-inducible CMV-LoxP-EGFP-LoxP-DsRed-P2A-porcine TP53^R167H^-T2A-human BRAF^V600E^ (CMV-LGL-DTB)^40^. The G^Cas9^ sequence in the plasmid was removed via double digestion with *SacI* and *BamHI*. Subsequently, the excised G^Cas9^ was replaced with the LoxP-flanked *EGFP* gene obtained from the CMV-LoxP-EGFP-pA-LoxP construct^50^. The HRAS^G12V^ sequence was cleaved using *EcoRI* and *MluI*, and the synthesized *AgeI* restriction enzyme site was added. Finally, XhoI-porcine TP53^R167H^-NruI and AgeI-human BRAF^V600E^-AgeI gene sequences synthesized by Synbio Technologies (NJ, USA) were integrated into the EcoRI-MluI and AgeI sites, respectively.

To increase the efficiency of integration into the genome, the SB transposon system was applied to CMV-LGL-DTB and porcine TYR-CreER^T2^. Briefly, the synthesized *ClaI* restriction enzyme site was added between *BglII* and *EcoRI* sites in the transposon element vector pT4/HB (#108352; Addgene, Watertown, MA, USA). The CMV-LGL-DTB cassette was obtained via *ClaI* digestion and incorporated into an identical site of the modified pT4/HB. Finally, the porcine TYR-CreER^T2^-bGH poly(A) cassette digested with *SphI* from the pcDNA3.1-pig GFAP-CreER^T2^ plasmid was inserted into an identical enzyme site in the pT4/HB-CMV-LGL-DTB plasmid vector.

### Transgenic donor cell construction

In total, 1 × 10^6^ pig fibroblasts were co-transfected with 0.5 µg transposase vector, pCMV(CAT)T7-SB100 (#34879, Addgene), and 4 µg CMV-LGL-DTB-pTYR-CreER^T2^ transposon oncogene cassette vector using a Neon Transfection System (Invitrogen) with a single 20 ms/1300 V pulse. Cells were subsequently transferred to complete medium. After 10–12 d, EGFP-positive cells were sorted using flow cytometry (FACS Aria II, BD Biosciences, Franklin Lakes, NJ, USA).

### Oocyte collection and in vitro maturation

Oocyte collection and in vitro maturation (IVM) were performed as previously described^57^. Porcine ovaries were obtained from a local slaughterhouse and transported to the laboratory within 1 h in 0.9% (v/v) NaCl solution at 37 °C. Cumulus-oocyte complexes (COCs) were collected from medium-sized (3–7 mm in diameter) follicles and washed with HEPES-buffered Tyrode’s medium containing 0.05% (w/v) polyvinyl alcohol (TLH-PVA). COCs were cultured in IVM medium consisting of TCM-199 (Gibco) supplemented with 0.6 mM cysteine, 0.91 mM sodium pyruvate, 10 ng/mL epidermal growth factor, 75 µg/mL kanamycin, 1 µg/mL insulin, and 10% (v/v) porcine follicular fluid. During the first 22 h of IVM, COCs were incubated in an IVM medium containing 10 IU/mL equine chorionic gonadotropin and 10 IU/mL human chorionic gonadotropin at 39 °C in a humidified 5% CO_2_ atmosphere. After 22 h, COCs were transferred to a hormone-free IVM medium and incubated for 20 h.

### SCNT, in vitro culture of porcine embryos, and embryo transfer

After 42 h of IVM, mature COCs were denuded via mechanical pipetting with 0.1% hyaluronidase. Metaphase II-stage (MII) oocytes were used for SCNT according to a previously reported method^58^. Briefly, MII oocytes were enucleated using a 16 µM micromanipulator glass pipette in calcium-free TLH containing 5 µg/mL cytochalasin B. Following enucleation, a donor cell was injected into the perivitelline space of enucleated oocytes. They were subsequently fused using an Electro Cell Fusion Generator (LF101; Nepa Gene, Chiba, Japan) with two pulses of 160 V/mm direct current for 60 µs in 2 mL of 260 mM mannitol solution containing 0.1 mM CaCl_2_ and 0.05 mM MgCl_2_. Fused SCNT embryos were cultured in porcine zygote medium (PZM) containing 0.4 µg/mL demecolcine and 6-dimethyl amino purine for 4 h (post-activation). Thereafter, the embryos were transferred to PZM drops covered with mineral oil for in vitro culture (IVC). SCNT was performed on day 0. On day 2, analysis of embryo cleavage was performed (1-, 2–3-, 4–5-, 6–8-cell, and fragmented embryos), and the embryos were transferred to new PZM drops. On day 4, the embryos were transferred to PZM drops containing 10% (v/v) FBS. Blastocyst formation was quantitatively evaluated (early, expanded, and hatched) 7 d after fusion. Jeju native pigs were used as surrogate mothers for embryo transfer by Cronex Inc. (Cheongju, South Korea). Midventral laparotomy was performed to visualize reproductive organs. The reconstructed embryos were transferred to surrogate oviducts. After 28 d, ultrasonography was used to confirm pregnancy.

### Derivation of nuclear transfer embryonic stem cell lines

After removing the zona pellucida using 0.5% protease (Sigma-Aldrich), SCNT blastocysts on day 6 were seeded onto mitomycin C-treated mouse embryonic fibroblast (MEF) feeder cells (5 × 10^4^ cells per cm^2^). Nuclear transfer embryonic stem cells (ntESCs) were cultured in FIW medium^59^ at 37 °C and 5% CO_2_. The FIW medium consisted of DMEM/F12, 10% KnockOut Serum Replacement (Gibco), 1× non-essential amino acids, 0.05 mM β-mercaptoethanol, 1% antibiotic-antimycotic, 10 ng/mL recombinant human fibroblast growth factor-basic (100-18B; Peprotech, Cranbury, NJ, USA), 1.5 µM IWR-1 (I0161; Sigma-Aldrich), and 0.3 µM WH-4-023 (S7565; Selleckchem). The FIW medium was changed daily. At 7–9 d after blastocyst seeding, primary colonies were passaged onto MEF feeder cells using TrypLE™ Express Enzyme (Gibco). Subsequently, porcine ntESC lines were cultured in FIW medium supplemented with 10 µM Y-27632 (S1049; Selleckchem, Houston, TX, USA), which was supplied only 24 h after subculture.

### Alkaline phosphatase staining

Alkaline phosphatase (AP) activity of ntESCs was detected using the NBT/BCIP chromogen solution (Roche)^58^. ntESCs were washed with Dulbecco’s phosphate-buffered saline (DPBS), fixed in 4% paraformaldehyde for 10 min at 25 °C, and washed thrice with Tris solution (0.1 M Tris, NaCl, pH 9.48). ntESCs were incubated in Tris solution supplemented with NBT/BCIP chromogen solution for 2 h and observed under a microscope.

### Karyotype analyses

Karyotyping of ntESCs using standard G-banding chromosomes and cytogenetic analysis were performed at the Korea Research of Animal Chromosomes (www.krach.co.kr, Korea).

### Embryoid body differentiation

ntESCs were dissociated using TrypLE™ Express Enzyme (Gibco) and cultured for 7 d on 35 mm low-attachment plates in DMEM (Gibco) containing 10% (v/v) FBS, 0.1% 2-mercaptoethanol (Gibco), 1% GlutaMAX (Gibco), 1% non-essential amino acids (Gibco), 1% antibiotic-antimycotic, and 10 µM Y-27632 (24 h only). After suspension culture, aggregated and spherical EBs were plated on 0.1% (w/v) gelatin-coated 8-well chamber slides (154534; Thermo Fisher Scientific, Waltham, MA, USA) for 1 week in the same medium and fixed with 4% paraformaldehyde for immunostaining.

### Cell culture

Primary fibroblasts were isolated from ear tissues of each piglet according to previously described methods^60^. Similarly, primary umbilical cord cells were isolated from individual piglet umbilical cords following the same protocol. The cells were maintained in DMEM (Gibco) supplemented with 10% (v/v) FBS, 1% GlutaMAX (Gibco), 1% non-essential amino acids (Gibco), 1% antibiotic-antimycotic, and 0.1% 2-mercaptoethanol.

### Genomic DNA and total RNA extraction

Genomic DNA was isolated from indicated cells using the Wizard® Genomic DNA Purification Kit (Promega) according to the manufacturer’s instructions. Transgenic pig tissues were homogenized using Bullet Blender (Next Advance, Troy, NY, USA) following the manufacturer’s instructions. Total RNA was extracted using TRIzol reagent (TaKaRa Bio, Inc.) according to the manufacturer’s instructions. Subsequently, 1 µg of total RNA was converted into complementary DNA (cDNA) using SuperScript IV VILO Master Mix (Thermo Fisher Scientific).

### PCR and gel electrophoresis

Genomic DNA was amplified in a 20 µL PCR reaction containing 1 U of PrimeSTAR® GXL DNA polymerase (TaKaRa Bio, Inc.), 1× GXL buffer, 2.5 mM deoxyribonucleoside triphosphate mix, and 5 pM of each gene-specific primer. PCR conditions were as follows: pre-denaturation at 95 °C for 5 min, 35 cycles of denaturation at 95 °C for 30 s, annealing at 58 °C for 30 s, extension at 68 °C for 1 min/1-kbp, followed by 68 °C for 5 min. The amplified PCR products were analyzed using gel electrophoresis at 100 V for 30 min on a 1% agarose gel with Midori Green DNA dye (NIPPON Genetics EUROPE, Düren, Germany). Gel images were obtained using the Davinchi-Gel Imaging System (Davinch-K, Seoul, Republic of Korea). All oligonucleotide primer sequences are listed in Supplementary Table 1.

Inverse PCR to identify a transgene integration locus was performed as described^61^. Briefly, 2 µg genomic DNA was digested with *XmaI* (Takara Bio Inc.) at 37 °C overnight. Then, restriction enzymes were inactivated at 75 °C for 20 min. 50 ng of digested DNA was self-ligated using T4 ligase (Enzynomics, Inc., Daejeon, Korea) at 4 °C overnight. The first-round of PCR was performed using forward primer, 5′-AGAAGTCGTGCTGCTTCATG-3′, and reverse primer, 5′-ACGAGAAGCGCGATCACATG-3′, and PrimeSTAR® GXL DNA Polymerase using the following thermal cycling conditions: 60 s at 98 °C; 35 cycles of 15 s at 98 °C, 15 s at 58 °C, and 450 s at 72 °C; followed by cooling at 4 °C. Then, the second-round PCR was performed using forward primer, 5′-GCTTATATAGACCTCCCACCG-3′, and reverse primer, 5′-GCATGGACGAGCTGTACAAG-3′, under PCR conditions identical to those used in first-round PCR. The third-round PCR was performed using forward primer, 5′-CCAAGTGGGCAGTTTACCGT-3′, and reverse primer, 5′-GCATGGACGAGCTGTACAAG-3′. PCR products were separated by DNA electrophoresis on a 1% agarose gel, and in-gel DNA extraction was performed using a gel extraction kit (Elpis Biotech Inc., Daejeon, Korea) according to the manufacturer’s instructions. Finally, the PCR product was sequenced using an ABI BigDye® terminator v3.1 Cycle Sequencing kit (Applied Biosystems, CA, USA) at Bionics Corp. (Seoul, Korea).

### Quantitative reverse-transcription PCR

Quantitative reverse-transcription PCR (qRT-PCR) was conducted using 2× SYBR Premix Ex Taq (TaKaRa Bio, Inc.) according to the manufacturer’s instructions. A qRT-PCR mixture composed of synthesized cDNA, 2× SYBR Premix Ex Taq, and 5 pmol of specific primers (Macrogen, Inc., Seoul, Republic of Korea) was analyzed on a CFX96 Touch Real-Time PCR Detection System (Bio-Rad, Hercules, CA, USA). The 2^−ΔΔCT^ method was employed to determine the relative expression levels^62^. The mRNA expression levels were normalized to those of *RN18S*. All primer sequences are listed in Supplementary Table 1.

### Electroporation

To validate recombination of oncogene cassette in vitro, pcDNA3.1-Cre-3NLS-puro was delivered to fibroblasts via Neon® Transfection System (Invitrogen, CA, USA), according to the manufacturer’s instructions. In detail, total 10 × 10^5^ cells were transfected with 5 µg of plasmid by electroporation (pulse voltage: 1,300 V; pulse width: 30 ms; 1 time). The cells were harvested after 72 h and used in further experiments.

### Western blotting

For western blotting, whole-cell extracts were prepared using radioimmunoprecipitation assay lysis buffer (LPS solution, Daejeon, Republic of Korea) composed of 150 mM NaCl, 1% NP-40, 0.1% sodium dodecyl sulfate (SDS), and 50 mM Tris (pH 7.4) containing 1 mM NaF, 1 mM Na_3_VO_4_, 1 mM β-glycerophosphate, 2.5 mM sodium pyrophosphate, and protease inhibitor (Roche, Indianapolis, IN, USA). Proteins were quantified using Bradford assay reagent (Bio-Rad, Hercules, CA, USA) according to the manufacturer’s instructions. Proteins were separated using 10% SDS-polyacrylamide gel electrophoresis and transferred onto polyvinylidene fluoride membranes (Pall Corporation, Tokyo, Japan). Membranes were blocked with 5% non-fat milk and incubated with the following antibodies: anti-BRAF^V600E^ (ab228461; Abcam, Cambridge, UK), anti-BRAF (sc-5284; Santa Cruz Biotechnology, Dallas, TX, USA), anti-p53 (sc-126; Santa Cruz Biotechnology), anti-pERK1/2 (9101; Cell Signaling Technology, Danvers, MA, USA), anti-ERK1/2 (9102; Cell Signaling Technology), anti-pAKT (Ser473, 9271; Cell Signaling Technology), anti-AKT (9272; Cell Signaling Technology), and β-actin (sc-47778; Santa Cruz Biotechnology). After washing, membranes were incubated with horseradish peroxidase-conjugated anti-IgG secondary antibody (31430, 31460; Invitrogen) and visualized using the SuperSignal West Pico Chemiluminescent Substrate (37578; Thermo Fisher Scientific). The antibodies used in this study are listed in Supplementary Table 2.

### Immunostaining

ntESCs or EBs were cultured in 8-well chamber slides on MEFs or slides coated with 0.1% gelatin for immunofluorescence staining, respectively. Cells were washed with DPBS, fixed with 4% paraformaldehyde at 25 °C for 10 min, permeabilized in 0.5% Triton X-100 for 10 min, and washed twice with DPBS. Cells were blocked in Immunofluorescence Blocking Buffer (12411; Cell Signaling) for 30 min at 25 °C and incubated overnight at 4 °C with primary antibodies: Oct-3/4 (sc-5279; Santa Cruz Biotechnology), Sox-2 (sc-365823; Santa Cruz Biotechnology), SSEA4 (ab16287; Abcam), β3-Tubulin (5568; Cell Signaling Technology), Desmin (MAB3430; Merck), Cytokeratin 17 (ab109725; Abcam), and GFP (ab6673; Abcam). Cells were subsequently washed thrice with DPBS for 5 min and incubated with the appropriate secondary antibodies at 25 °C for 1 h. After washing thrice with DPBS, nuclei were stained with Hoechst-33342 for 10 min.

To investigate the histological characteristics of tissue samples, obtained tissues were embedded in paraffin, sectioned (4 μm thickness), and placed on glass slides. Following deparaffinization and hydration, tissue slides were depigmented in 3% hydrogen peroxide (Merck, Rahway, NH, USA) at 65 °C for 2 h. Thereafter, tissue sections were boiled for 20 min in sodium citrate buffer (pH 6.0) for antigen retrieval; the buffer contained 10 mM sodium citrate (SAMCHUN Chemical, Seoul, Republic of Korea). Tissue slides were permeabilized with PBS supplemented with 3% Triton X-100 and blocked with 3% bovine serum albumin (BSA) in PBS for 1 h at 25 °C. The samples were stained with antibodies against Ki67 (NCL-Ki67; Leica Biosystems, Wetzlar, Germany), BRAF^V600E^ (ab228461; Abcam), MITF (AF5769; R&D Systems, Minneapolis, MN, USA), and DsRed (sc-390909; Santa Cruz Biotechnology) overnight at 4 °C, washed thrice for 5 min each in ice-cold 1% BSA in PBS, and incubated with Alexa 488 conjugated (A21202; Invitrogen), Alexa 594 conjugated (A11012; Invitrogen), or Alexa 647 conjugated (A32849; Invitrogen) secondary antibodies for 2 h. After staining with DAPI (1:1000, D9542; Sigma-Aldrich), the slides were mounted in a mounting solution (P36930; Invitrogen). Fluorescence was detected using a confocal laser scanning microscope (LSM800; Carl Zeiss, Oberkochen, Germany). Images were analyzed using the ImageJ software (http://imagej.nih.gov). The quantification of Ki67-positive cells was performed by analyzing counting Ki67 expressing cells among DAPI-stained cells in four different regions of each tissue. The relative fluorescence intensity was compared by analyzing mean intensity of BRAF^V600E^, MITF, and DsRed staining in four different regions of each tissue.

Embryo immunostaining was performed as previously reported^63^. Briefly, the embryos were fixed with 4% paraformaldehyde in PBS for 30 min at 25 °C and permeabilized with 0.5% Triton X-100 for 1 h at 25 °C. Thereafter, embryos were treated with Image-iT™ FX Signal Enhancer (Invitrogen) for 30 min at 25 °C, blocked in Immunofluorescence Blocking Buffer (12411; Cell Signaling), and incubated with primary antibodies diluted with Blocking Buffer at 4 °C overnight. The embryos were washed thrice for 5 min with 0.1% Tween 20 and 0.01% Triton X-100 in 0.1% PVS (TTVS) at 25 °C. Subsequently, they were incubated with secondary antibodies and Phalloidin-iFluor 594 Reagent (F-actin, ab176757; Abcam) for 1 h 30 min at 25 °C. After washing thrice with TTVS, samples were counterstained with Hoechst-33342 for 10 min and mounted on glass slides in a mounting solution (S36967; Invitrogen). Fluorescent signals were detected under a confocal laser-scanning microscope (Leica STELLARIS 5). The antibodies used in this study are listed in Supplementary Table 2.

### 4-OHT treatment of porcine skin

An intradermal injection of 4-OHT (70% Z-isomer, H6278; Sigma-Aldrich) was administered to activate the CreER^T2^-inducible system. The appropriate injection needle and volume were determined according to previously reported methods^64^. Before 4-OHT treatment, 3-month-old pigs were anesthetized, and the left lateral loin regions were depilated using a hair clipper. The depilated area was disinfected with iodized povidone. The loins of each pig were divided into twelve 5 × 5 cm quadrants. A solution of 4-OHT was diluted to 20 mg/mL in ethanol. Intradermal injection of 100 µL 4-OHT per site was administered to clipped loins using a 26 G needle (1 mL/cc). A single pig was administered intradermal injections at four sites with solvent (control) and eight sites with 4-OHT. For quantification of skin pigmentation, the solvent or 4-OHT injection sites were defined as regions of interest (ROI) at 45 days post-treatment. The pigmentation was then quantified using ImageJ software, applying the following formula: ROI Integrated Density-(ROI Area*Background Mean).

### Hematoxylin and eosin (H&E) staining

Tissues from WT and TB-pTYR::Cre pigs were fixed in 10% buffered formaldehyde. Subsequently, the samples were paraffin-embedded and sectioned at a thickness of 3 μm. Following deparaffinization, the sections were stained with H&E.

### IVF

Mature oocytes were used for IVF after IVM. Fifteen oocytes were transferred to a modified Tris-buffered medium (mTBM) drop. Fresh liquid (Xperm-V; Darby Genetics, Inc., Anseong, South Korea) or TB-pTYR::Cre P#3 semen was diluted to a final concentration of 5 × 10^5^ spermatozoa/mL per drop and cocultured with oocytes for 20 min at 39 °C in a 5% CO_2_ humidified atmosphere. Spermatozoa that adhered to the surface of oocytes were detached via gentle pipetting, transferred to a new mTBM drop, and incubated for 5 h at 39 °C under the same atmospheric conditions. Thereafter, the embryos were subjected to the IVC procedure.

### Statistical analysis

The data are presented as the mean ± SEM. Differences between groups were analyzed using a two-tailed Student’s *t*-test. Statistical analyses were performed using SPSS (version 21.0; SPSS Inc., Chicago, IL, USA). Differences were considered statistically significant at *p < 0.05, **p < 0.01, ***p < 0.001, and ****p < 0.0001.

## Electronic supplementary material

Below is the link to the electronic supplementary material.

Supplementary Material 1

Supplementary Material 2

## Data Availability

Data is provided within the manuscript or supplementary information files.
