# Supplementary material for: Generation of a genetically engineered porcine melanoma model featuring oncogenic control through conditional Cre recombination
Source: Sci Rep. 2025 Jan 10;15:1616. doi: 10.1038/s41598-024-82554-w (PMC11724099; doi:10.1038/s41598-024-82554-w)
Supplement: Supplementary file 1 — Supplementary Material 1 [file 41598_2024_82554_MOESM1_ESM.docx]

**Supplementary Information**

**Generation of a genetically engineered porcine melanoma model featuring oncogenic control through conditional Cre recombination**

Dongjin Oh^1,2†^, Nayoung Hong^3,4†^, Kiyoung Eun^3,4,5†^, Joohyeong Lee^1,2,6^, Lian Cai^1,2^, Mirae Kim^1,2^, Hyerin Choi^1,2^, Ali Jawad^1,2^, Jaehyung Ham^1,2^, Min Gi Park^3,4^, Bohye Kim^7^, Sang Chul Lee^8^, Changjong Moon^7^, Hyunggee Kim^3,4*^, Sang-Hwan Hyun^1,2,9,10*^

^1^Laboratory of Veterinary Embryology and Biotechnology (VETEMBIO), Veterinary Medical Center and College of Veterinary Medicine, Chungbuk National University, Cheongju, Republic of Korea

^2^Institute of Stem Cell and Regenerative Medicine (ISCRM), Chungbuk National University, Cheongju, Republic of Korea

^3^Department of Biotechnology, College of Life Sciences and Biotechnology, Korea University, Seoul 02841, Republic of Korea

^4^Insitute of Animal Molecular Biotechnology, Korea University, Seoul 02841, Republic of Korea

^5^Department of Hematology and Medical Oncology, Winship Cancer Institute of Emory, Emory University School of Medicine, Atlanta, GA 30322, United States of America

^6^Department of Companion Animal Industry, Semyung University, Jecheon 27136, Republic of Korea

^7^Department of Veterinary Anatomy and Animal Behavior, College of Veterinary Medicine and BK21 FOUR Program, Chonnam National University, Gwangju, Korea

^8^Cronex Inc., Cheongju 28174, Korea

^9^Vet-ICT Convergence Education and Research Center (VICERC), Chungbuk National University, Cheongju, Republic of Korea

^10^Chungbuk National University Hospital, Cheongju, Republic of Korea

^†^These authors contributed equally: Dongjin Oh, Nayoung Hong, and Kiyoung Eun

***Corresponding authors:** Hyunggee Kim, hg-kim@korea.ac.kr; Sang-Hwan Hyun, shhyun@cbu.ac.kr

**The Supplementary Information includes:**

Supplementary Figures 1 to 9

Supplementary Tables 1 to 2


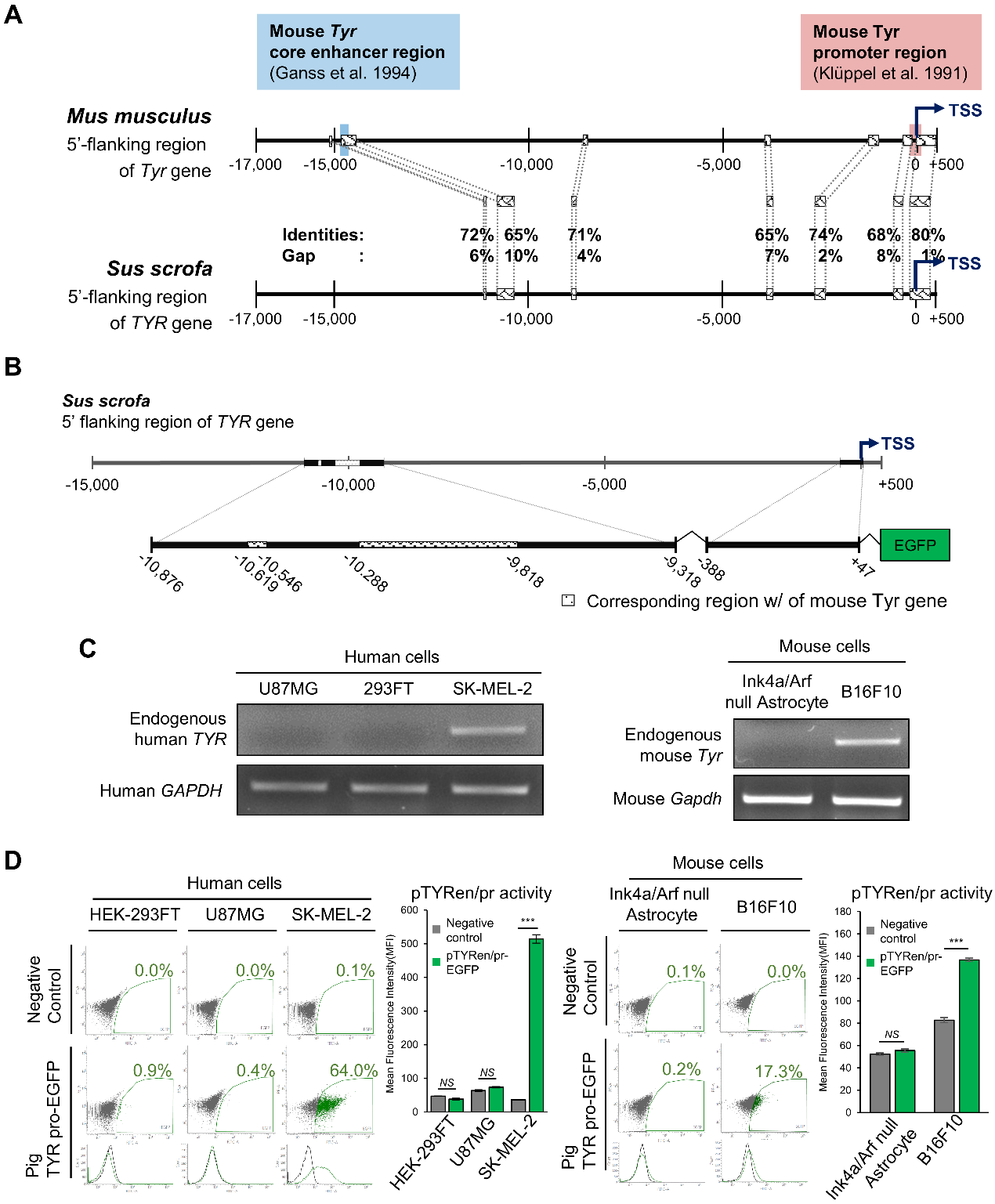


**Supplementary Figure 1.** Construction of melanoma-inducing system in porcine model. (**A**) Schematic diagram of the 5′-flanking region of murine *Tyr* and porcine *TYR*. (**B**) Schematic diagram of the porcine *TYR* enhancer and promoter (p*TYR*en/pr)-EGFP construct. (**C**) Expression of human *TYR* in human glioblastoma (U87MG), human embryonic kidney-293FT, and melanoma (SK-MEL-2) cell lines and mouse *Tyr* in Ink4a/Arf^-/-^ astrocytes and melanoma cells (B16F10). (**D**) p*TYR*en/pr activity in HEK-293FT, U87MG, SK-MEL-2, Ink4a/Arf^-/-^ astrocytes, and B16F10. Data are presented as mean ± SEM, n = 3. The data were analyzed using Student’s t-test. ^***^*p* < 0.001. NS, no significance.


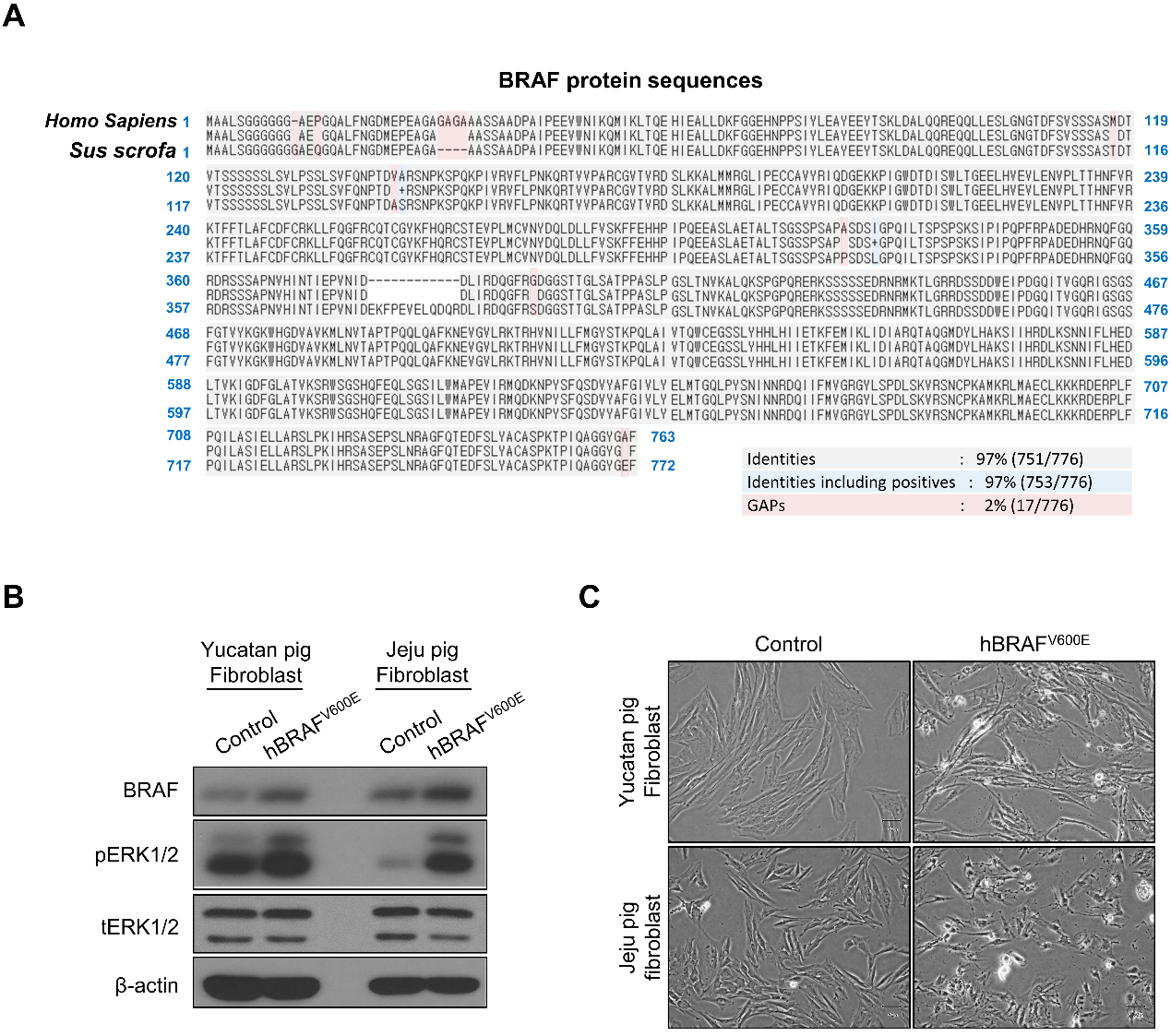


**Supplementary Figure 2.** Functional validation of human BRAF^V600E^ in porcine fibroblasts. (**A**) Homology comparison analysis of human BRAF and porcine BRAF proteins. (**B**) Western blot analysis of human BRAF^V600E^ (hBRAF^V600E^)-induced ERK phosphorylation in porcine fibroblasts. (**C**) Representative images of cellular morphology after hBRAF^V600E^ transduction in porcine fibroblasts.


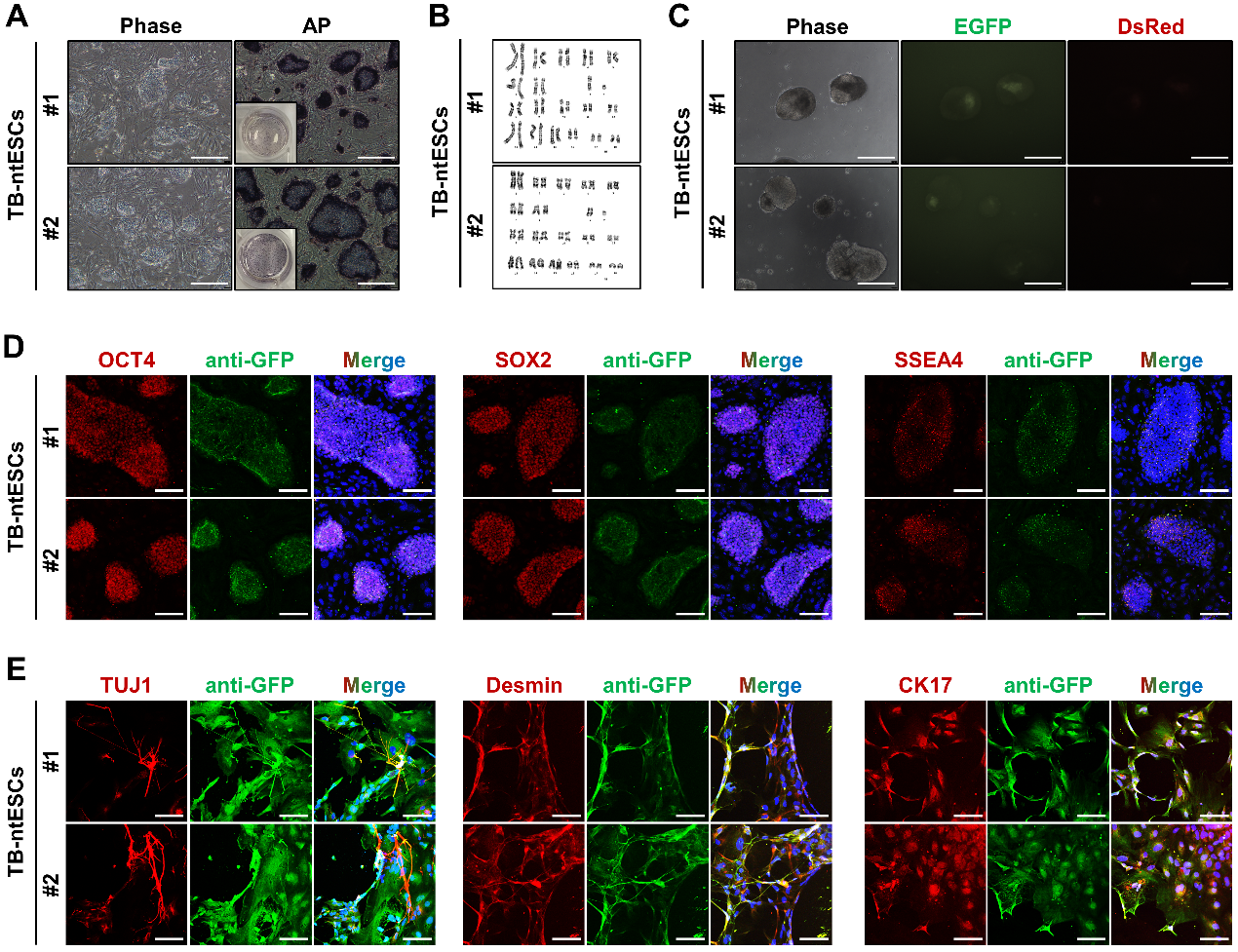


**Supplementary Figure 3.** Characterization of two TB-pTYR::Cre SCNT-derived porcine embryonic stem cells (TB-ntESCs). (**A**) Detection of alkaline phosphatase (AP) activities in two TB-ntESCs. Scale bar, 300 μm. (**B**) Normal chromosomes (36 + XY) were observed in each TB-ntESC line. (**C**) Representative images of embryoid bodies (Day 7). Scale bar, 300 μm. (**D**) Immunofluorescence staining of the pluripotency factors OCT4, SOX2, and surface marker SSEA4. (**E**) Immunofluorescence staining of the differentiation marker β3-tubulin (TUJ1; ectoderm), Desmin (mesoderm), and Cytokeratin 17 (CK17; endoderm) in embryoid bodies. Scale bar, 100 μm.


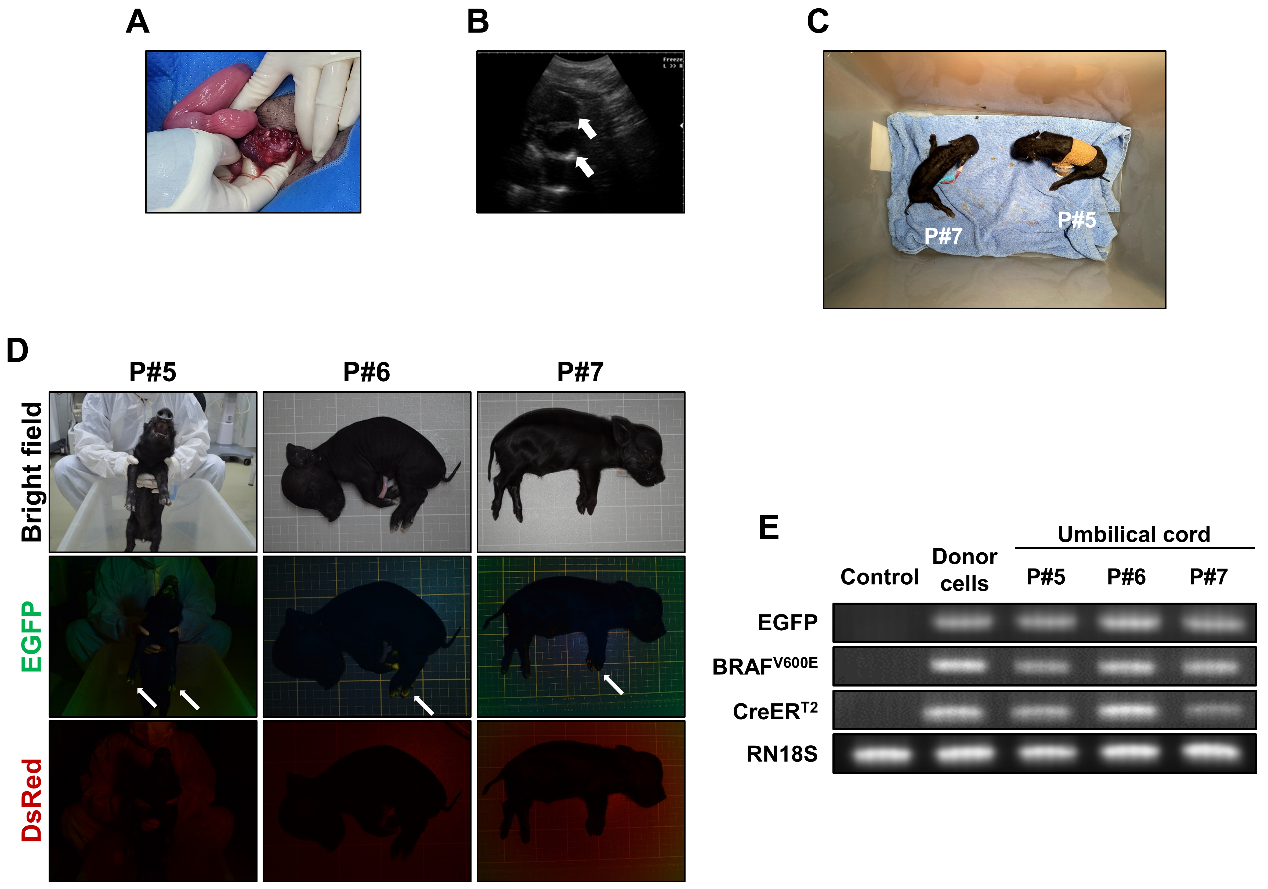


**Supplementary Figure 4.** Production of transgenic piglets with TB-pTYR::Cre transgene constructs. (**A**) Image of surrogate GY-18’s ovary during embryo transfer. (**B**) Image of pregnancy diagnosis from surrogate GY-18 on day 28 after embryo transfer. Bold arrow, embryonic sac. (**C**) Image of TB-pTYR::Cre piglets. P, pig. (**D**) Images show EGFP expression detected in hooves under UV light in each TB-pTYR::Cre piglet. Thin arrow, EGFP expression. (**E**) PCR was performed using genomic DNA isolated from the umbilical cord of TB-pTYR::Cre piglets. Control, normal pig fetal fibroblasts. *RN18S* was used as a control.


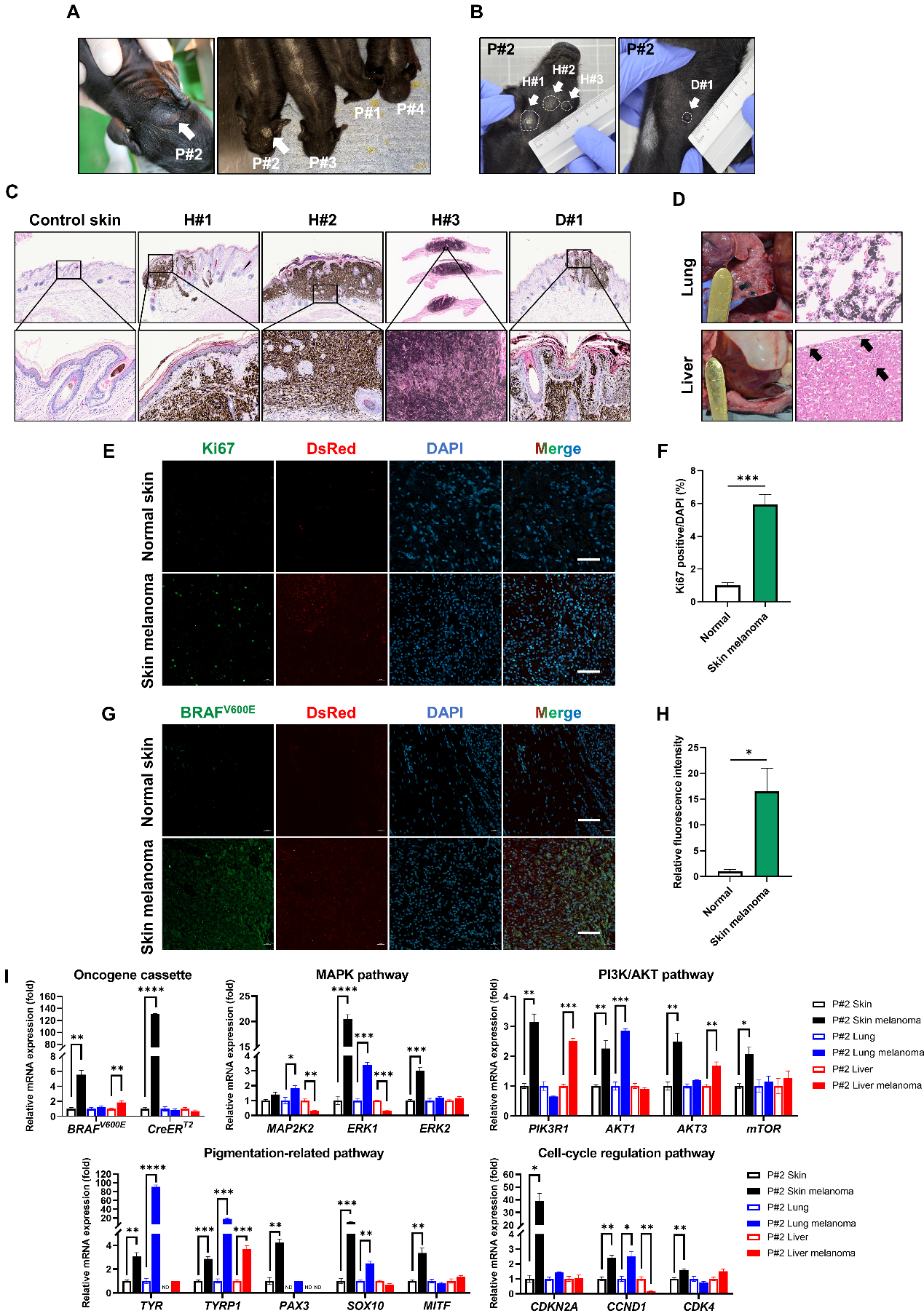


**Supplementary Figure 5.** Characterization of unintended melanomas in TB-pTYR::Cre P#2. (**A**) Images of an unintended head tumor from TB-pTYR::Cre P#2 on day 1 post-birth. (**B**) Images of unintended head and dorsal tumors from TB-pTYR::Cre upon euthanasia (day 14 post-birth). H, head; D, dorsal. White arrow, tumors. (**C**) Representative histology of unintended melanomas with H&E staining from the head and dorsal regions of TB-pTYR::Cre P#2. Scale bar, 300 μm (up) and 60 μm (down). (**D**) Representative histology with H&E staining from liver and lungs of TB-pTYR::Cre P#2. Black arrow, melanosis in liver. (**E**) Representative immunofluorescence image of Ki67 and DsRed expression in normal skin and pigmented region of the TB-pTYR::Cre P#2 skin melanoma. Scale bar, 150 μm. (**F**) Percentage of Ki67-positive cells in normal skin and pigmented region of the TB-pTYR::Cre P#2 skin melanoma. Data are presented as mean ± SEM, n = 4. The data were analyzed using Student’s t-test. ^***^*p* < 0.001. (**G**) Representative immunofluorescence image of BRAF^V600E^ and DsRed expression in normal skin and pigmented region of TB-pTYR::Cre P#2 skin. Scale bar, 150 μm. (**H**) Quantification of BRAF^V600E^ in normal skin and pigmented region of the TB-pTYR::Cre P#2 skin melanoma. Data are presented as mean ± SEM, n = 4. The data were analyzed using Student’s t-test. ^*^*p* < 0.05. (**I**) Quantification of mRNA expression of oncogene cassette (*BRAF^V600E^* and *CreER^T2^*), MAPK pathway-related genes (*MAP2K2*, *ERK1*, and *ERK2*), PI3K/AKT pathway-related genes (*PIK3R1*, *AKT1*, *AKT3*, and *mTOR*), pigmentation-related genes (*TYR*, *TYRP1*, *PAX3*, *SOX10*, and *MITF*), and cell-cycle regulation genes (*CDKN2A*, *CCND1*, and *CDK4*) using qRT-PCR in each tissue. Data are presented as mean ± SEM, n = 3. The data were analyzed using Student’s t-test. ^*^*p* < 0.05, ^**^*p* < 0. 01, ^***^*p* < 0.001, and ^****^*p* < 0.0001.

**
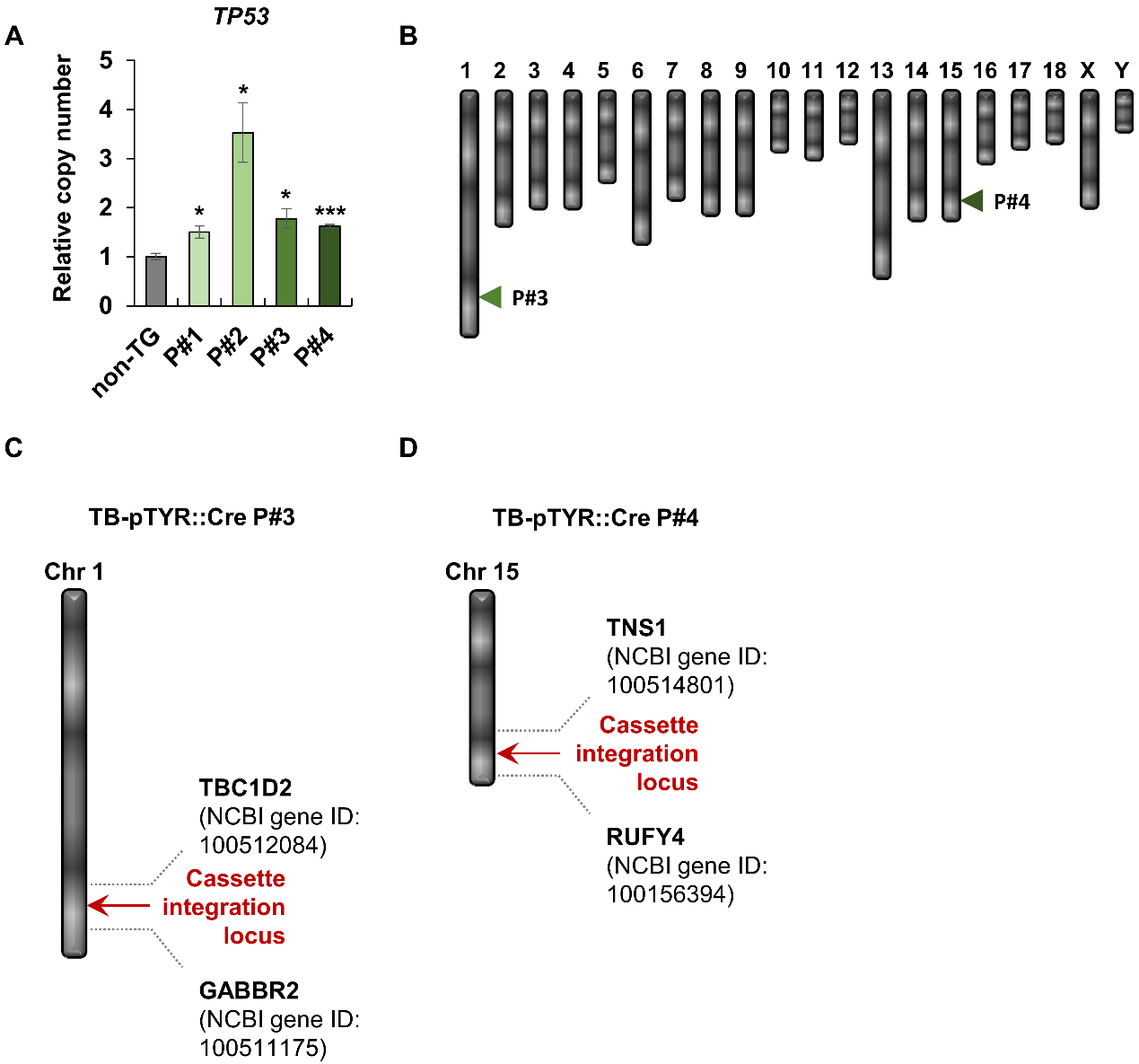
**

**Supplementary Figure 6.** Validation of integration locus of transgene in transgenic (TG) genome. (**A**) Copy number of TP53 in non-TG and four TG pig genome identified by quantitative PCR. The data was analyzed with 2^-ΔΔCt^ method to obtain relative copy number alteration compared to non-TG pig cells. Porcine *RN18S* was used as a reference gene. (**B**) Schematic diagram of porcine chromosome and integration locus (arrowhead) of transgene in each TG pig. (**C** and **D**) Schematic diagram of transgene integration locus and neighboring genes in TB-pTYR::Cre (**C**) P#3 and (**D**) P#4.


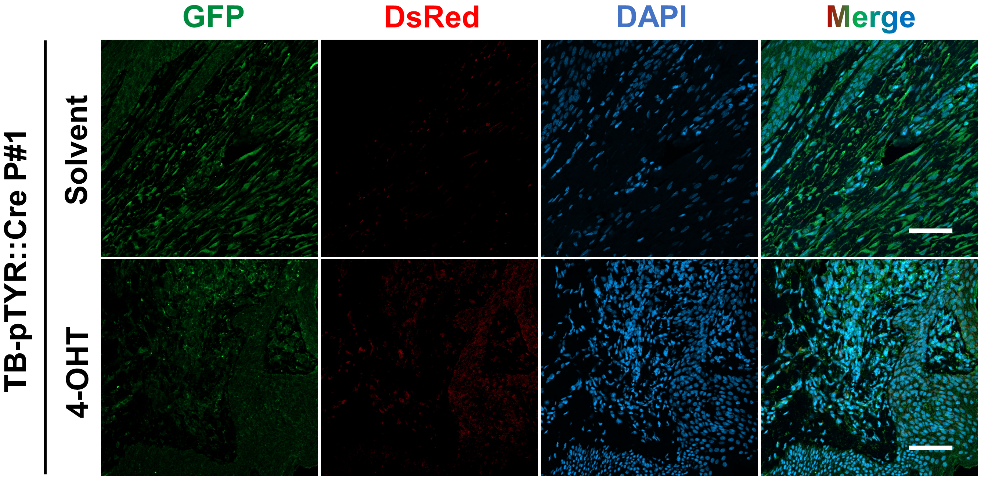


**Supplementary Figure 7.** Representative immunofluorescence image of GFP and DsRed expression in TB-pTYR::Cre P#1 skin following solvent and 4-OHT treatment. Scale bar, 150 μm.


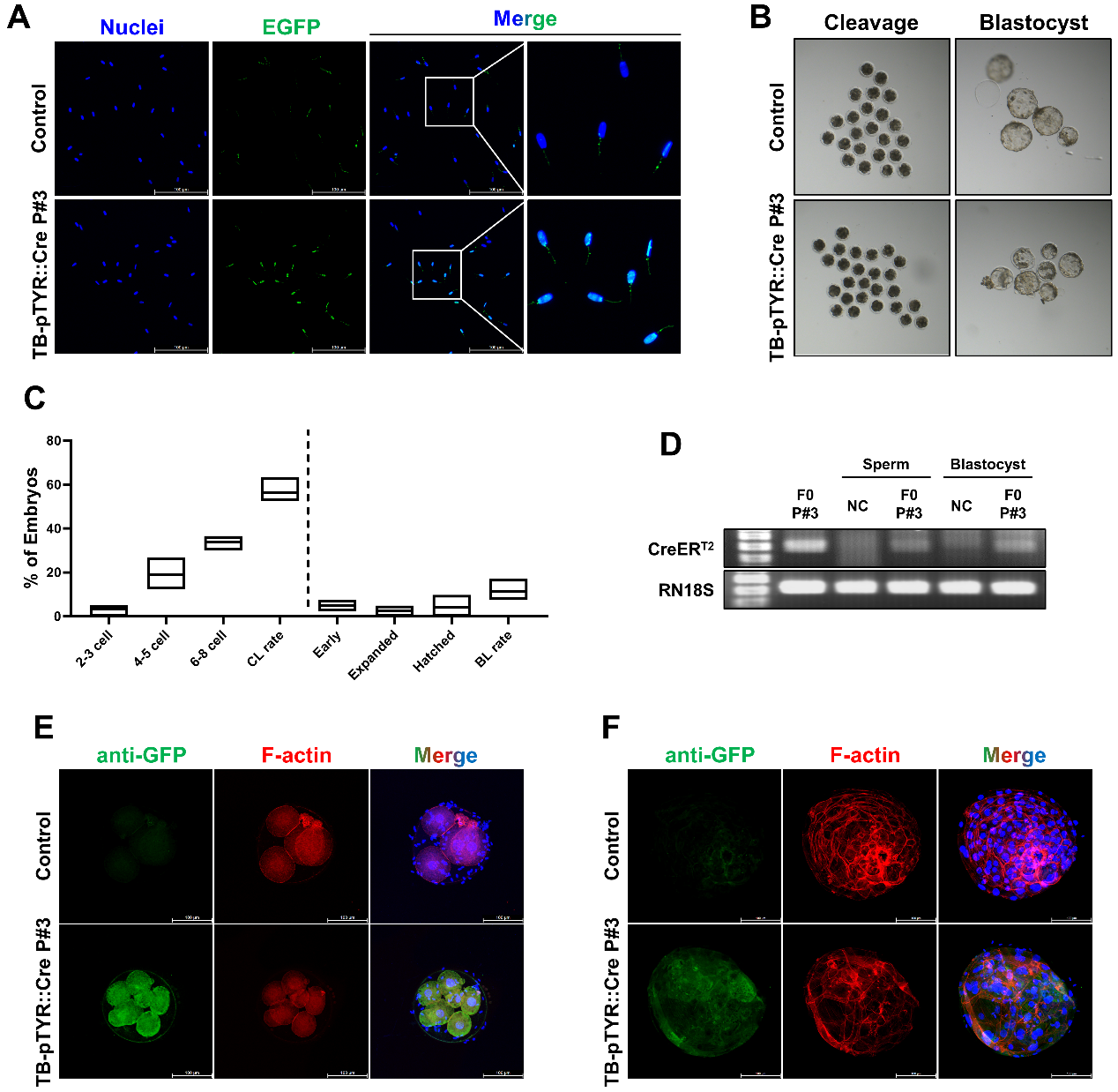


**Supplementary Figure 8.** Characterization of TB-pTYR::Cre P#3-derived sperm and evaluation of *in vitro* fertilized (IVF) embryo development. (**A**) Representative images showing EGFP expression in sperm from TB-pTYR::Cre P#3. Scale bar, 100 μm. (**B**) Bright field images of day 2 embryos and day 7 blastocysts after IVF. (**C**) Graphical representation of the cleavage pattern and blastocyst formation rate of IVF embryos using TB-pTYR::Cre P#3 sperm. The value represents mean ± SEM. CL, cleavage; BL, blastocyst. (**D**) PCR was performed using genomic DNA obtained from the sperm and a single IVF blastocyst of TB-pTYR::Cre P#3. (**E** and **F**) Representative laser scanning confocal microscopy images of porcine (**E**) cleavages and (**F**) blastocysts labeled with anti-GFP (green), F-actin (red), and Hoechst 33342 (blue) after IVF. Scale bar, 100 μm.


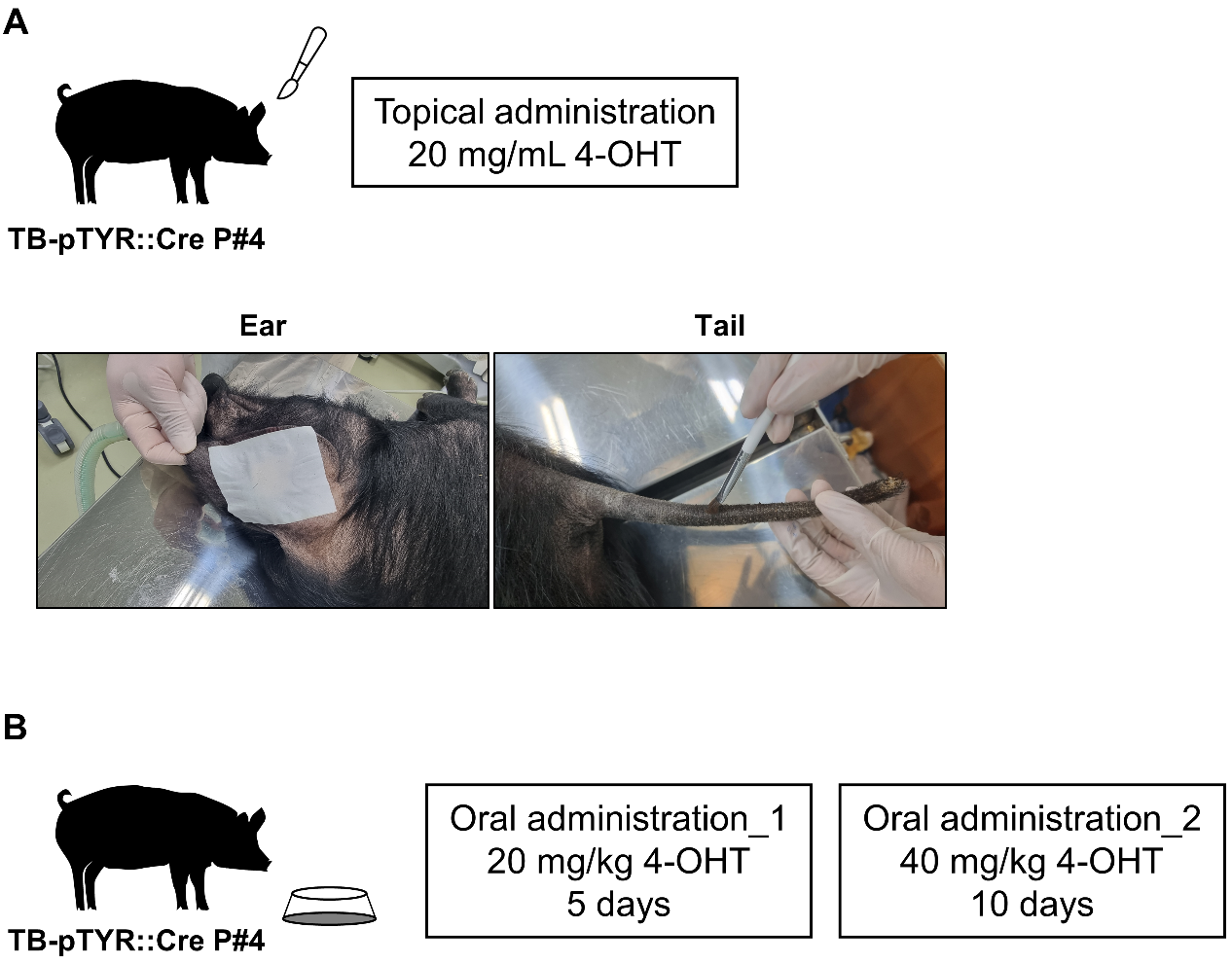


**Supplementary Figure 9.** Induction of the TB-pTYR::Cre construct system after 4-hydroxytamoxifen (4-OHT) treatment. (**A**) Scheme of 4-OHT topical administration. Representative images of ear and tail topically treated with 4-OHT. (**B**) Scheme of 4-OHT oral administration.

Supplementary Table 1. Primers used in this study.

| Target sequence | Primer sequence (5′–3′) | |
| --- | --- | --- |
| Oncogene cassette | F | CAAGTACGCCCCCTATTGAC |
|  | R | GACGATGATTTCCCCGACAA |
| CMV-to-DsRed | F | CAAGTACGCCCCCTATTGAC |
|  | R | TCCACGTAGTAGTAGCCGGG |
| *CreER^T2^* | F | TCGCAAGAACCTGATGGACA |
|  | R | CGCCGCATAACCAGTGAAAC |
| *hBRAF^V600E^* | F | GCATGGATTACTTACACGCCA |
|  | R | CGAGAATTTGGGGAAAGAGTGGT |
| *RN18S* | F | CGCGGTTCTATTTTGTTGGT |
|  | R | AGTCGGCATCGTTTATGGTC |
| *EGFP* | F | GCTGACCCTGAAGTTCATCT |
|  | R | TCGTCCTTGAAGAAGATGGT |
| *MAP2K2* | F | GCTTCTATGGGGCCTTCTAC |
|  | R | GTTGGATGGCTTCACATCTC |
| *ERK1* | F | ATCACAGTGGAGGAAGCACT |
|  | R | GAGGCATCTGTCCAGGTTAG |
| *ERK2* | F | AGTCCATCGACATCTGGTCT |
|  | R | GAGCTTTGGAGTCAGCATTT |
| *PIK3R1* | F | CCACTACCGGAATGAATCTC |
|  | R | TTCCTGGGAAGTACGGGTAT |
| *AKT1* | F | CTACAACCAGGACCACGAGA |
|  | R | CTCATACACATCCTGCCACA |
| *AKT3* | F | TTTCCATTTGTCGAGAGAGC |
|  | R | TGCATCTGTGATCCCTTCTT |
| *mTOR* | F | CACTCGCTCTTTAGCAGTCA |
|  | R | ACCAGTGACCTCCATAGCAT |
| *TYR* | F | CAGATGCCTCTCAAAGCAGT |
|  | R | TGTAAGATTCCCGGTTGTGT |
| *TYRP1* | F | GTACCACCATTGAGGCTTTG |
|  | R | GCATGTCCTGTTGAAGAACC |
| *PAX3* | F | GAAACACCGTACCCTCAGTG |
|  | R | GTCGATGTCAGAGCCTTCAT |
| *SOX10* | F | TGCTGAACGAGAGTGACAAG |
|  | R | TCTTGTAGTGGGCCTGGAT |
| *MITF* | F | CACCATCACCTTCAACAACA |
|  | R | ATGCTCATACTGCTCCTTCG |
| *CDKN2A* | F | CGCCGTCTCTTGATTACTGT |
|  | R | TCATGACCTGGTCTAGGATG |
| *CCND1* | F | CTCGAAGATGAAGGAGACCA |
|  | R | GTGTTTGCGGATGATCTGTT |
| *CDK4* | F | TGGAAACTCTGAAGCTGACC |
|  | R | CGCTTGTGTGGGTTAAAAGT |

F: Forward, R: Reverse.

Supplementary Table 2. Antibodies used in this study.

| Reagent | Source | Cat # | Dilution |
| --- | --- | --- | --- |
| Mouse monoclonal anti-BRAF^V600E^ | Abcam | ab228461 | 1:500 |
| Mouse monoclonal anti-BRAF | Santa Cruz Biotechnology | sc-5284 | 1:500 |
| Mouse monoclonal anti-p53 | Santa Cruz Biotechnology | sc-126 | 1:1000 |
| Rabbit anti-pERK1/2 | Cell Signaling Technology | 9101 | 1:500 |
| Rabbit anti-ERK1/2 | Cell Signaling Technology | 9102 | 1:500 |
| Rabbit anti-pAKT (Ser473) | Cell Signaling Technology | 9271 | 1:500 |
| Rabbit anti-AKT | Cell Signaling Technology | 9272 | 1:500 |
| Mouse monoclonal anti-β-actin | Santa Cruz Biotechnology | sc-47778 | 1:10000 |
| Rabbit monoclonal anti-Ki67 | Leica Biosystems | NCL-Ki67 | 1:200 |
| Goat polyclonal anti-MITF | R&D systems | AF5769 | 1:200 |
| Mouse monoclonal anti-DsRed | Santa Cruz Biotechnology | sc-390909 | 1:200 |
| Mouse monoclonal anti-Oct-3/4 | Santa Cruz Biotechnology | sc-5279 | 1:200 |
| Mouse monoclonal anti-Sox-2 | Santa Cruz Biotechnology | sc-365823 | 1:100 |
| Mouse monoclonal anti-SSEA4 | Abcam | ab16287 | 1:200 |
| Rabbit monoclonal anti-β3-Tubulin | Cell Signaling Technology | 5568 | 1:200 |
| Mouse monoclonal anti-Desmin | Merck | MAB3430 | 1:200 |
| Rabbit monoclonal anti-Cytokeratin 17 | Abcam | ab109725 | 1:200 |
| Goat polyclonal anti-GFP | Abcam | ab6673 | 1:200 |
| Phalloidin-iFluor 594 Reagent | Abcam | ab176757 | 1:500 |
| Goat anti-Mouse IgG (H+L) Cross-Adsorbed Secondary Antibody, Alexa Fluor™ 594 | Invitrogen | A11005 | 1:400 |
| Donkey anti-Mouse IgG (H+L) Highly Cross-Adsorbed Secondary Antibody, Alexa Fluor™ 488 | Invitrogen | A21202 | 1:400 |
| Goat anti-Rabbit IgG (H+L) Cross-Adsorbed Secondary Antibody, Alexa Fluor™ 594 | Invitrogen | A11012 | 1:400 |
| Donkey anti-Goat IgG (H+L) Highly Cross-Adsorbed Secondary Antibody, Alexa Fluor™ Plus 647 | Invitrogen | A32849 | 1:400 |
| Goat anti-Mouse IgG (H+L) Secondary Antibody, HRP | Invitrogen | 31430 | 1:5000 |
| Goat anti-Rabbit IgG (H+L) Secondary Antibody, HRP | Invitrogen | 31460 | 1:5000 |
